# Harvest time differentially shapes flavor compounds in red and white strawberry cultivars (*Fragaria × ananassa* ‘Jiandehong’ and ‘Tianshi’) and their wines via cultivar-specific metabolic responses

**DOI:** 10.1016/j.fochx.2026.104622

**Published:** 2026-10-13

**Authors:** Hongmin Liang, Cong Wang, Xiaoyu Yu, Yanxia Zhao, Jiabo Sun

**Affiliations:** a Institute of Leisure Agriculture, Shandong Academy of Agricultural Sciences, Jinan 250100, China; b College of Food Science and Engineering, Shandong Agriculture and Engineering University, Jinan 250100, China

**Keywords:** Strawberry (*Fragaria × ananassa*), Harvest time, Red and white cultivars, Flavor compounds, Strawberry wine

## Abstract

This study investigated the interactive effects of harvest time (January vs. March) and strawberry cultivar (red JDH; white TS) on fruit and wine quality. Cultivar × harvest interactions were significant for most parameters. JDH accumulated more sugars and acids in January, whereas TS maintained stable sugar-acid balance. March harvest was associated with higher phenolics and antioxidant capacity in both cultivars and wines, but color changes occurred only in red wines. JDH was rich in ellagic acid; January TS had high catechin and vanillin. Total volatiles were higher in January, while esters were enriched in March JDH and its wines. *E*-tongue sourness response correlated with total acid and pH, and astringency response with catechin and p-coumaric acid. White TS wines showed minimal color variation despite E-tongue responses and volatile changes. These findings reveal cultivar-specific metabolic buffering and partial retention of harvest time-imprinted signatures after fermentation, supporting white strawberry oenological potential.

## 1 Introduction

Strawberry (*Fragaria × ananassa*) is valued for its distinctive flavor and rich nutrition, yet its high moisture content (approximately 90%) and active metabolic activity limit fresh shelf life to about one week (Salazar-Orbea et al., 2021). Fermentation into low-alcohol strawberry wine (STw) extends raw material utilization while retaining bioactive components (Bezerra et al., 2024). However, STw often suffers from unstable aroma, poor stability, and inconsistent color, largely owing to raw-fruit variability co-regulated by cultivar and environmental conditions.

In China, greenhouse cultivation spans from November to April, with marked variation in temperature, humidity, and light. Temperature critically influences quality and a large diurnal temperature variation favors soluble solids accumulation (Khammayom et al., 2022), while elevated temperatures promote flavonoids and anthocyanins synthesis (Balasooriya et al., 2019). Consequently, nutritional and functional traits fluctuate with harvest time (HAR_T_), a seasonal variability that constrains China's fruit wine industry (Huang et al., 2022). Current research has largely focused on cultivar selection, yeast strains, and aging (Lan et al., 2024; Wang et al., 2025), or on flowering, yield, and postharvest storage (Meighani & Salehi Sarbijan, 2025; Rivero et al., 2022), with few studies addressing how seasonal variation affects intrinsic composition and processing adaptability (Simkova et al., 2023). In practice, raw material cost often dominates decisions, whereas differences among harvest times or cultivars, especially the emerging white strawberries, are frequently overlooked.

This study investigates the interactive effects of HAR_T_ and cultivar on processing adaptability. Red and white strawberries harvested in January and March were evaluated for physicochemical properties, color, organic acids, phenolics, antioxidant activity, volatiles, and *E*-tongue taste responses of in fruits and wines. Multivariate correlation analysis was used examined associations among metabolites, E-tongue signals, and key volatiles. The objective is to clarify how HAR_T_ modulates cultivar-specific metabolic profiles, supporting HAR_T_ and cultivar-specific utilization. Although white strawberry cultivars have been included in earlier winemaking studies (e.g., Lan et al., 2024), systematic evaluation of their oenological potential, particularly cultivar-specific metabolic responses and processing adaptability across harvest times, remains rare. Focusing on the HAR_T_ and cultivar interaction, this study provides an integrated compositional characterization of white strawberry wine and offers preliminary evidence for white strawberry germplasm utilization in winemaking.

## 2 Materials and methods

### 2.1 Materials and reagents

Commercial active dry yeast *Saccharomyces cerevisiae* EC-1118 and pectinase Lafazym extract (500 g) were purchased from Lallemand Inc. (Quebec, Canada). Twelve organic acid standards (oxalic, tartaric, malic, lactic, acetic, fumaric, citric, maleic, succinic, quinic, shikimic, malonic) and twenty-one phenolic standards (catechol, syringaldehyde, vanillin, o−/p-coumaric acids, kaempferol, catechin, p-hydroxybenzoic acid, rutin, ferulic/syringic/vanillic/benzoic acids, caffeic/quinic/chlorogenic/ gallic/sinapic acids, protocatechuic acids, protocatechualdehyde, epicatechin) were obtained from Shanghai Yuanye Biotechnology Co., Ltd. and Zhenzhun Biotechnology Co., Ltd. (Shanghai, China). HPLC-grade formic acid, acetonitrile, and methanol were from Thermo Fisher Scientific (Waltham, MA, USA) and Merck KGaA (Darmstadt, Germany). 4-methyl-2-pentanol (internal standard, IS) and n-ketone mixtures (C4–C9) were purchased from Sigma-Aldrich (St. Louis, MO, USA).

### 2.2 Preparation of strawberries and wines

Red strawberry cultivar ‘Jiandehong’ (JDH) and white cultivar ‘Tianshi’ (TS) were harvested in January (low-temperature winter stage) and March (warming spring stage) of 2025 at the Licheng Experimental Base (36°30′16” N, 117°13′23″ E), Shandong, China. Greenhouse temperature and relative humidity were recorded by S21A-2100 sensors (Pengyun IoT platform) (Table S1). Four treatments of JDH-1, JDH-3, TS-1, and TS-3 designated the JDH and TS cultivars harvested in January and March, respectively. Uniform field management (irrigation, fertilization, pruning and pest control) was applied throughout cultivation. At each harvest, only fully ripe fruits were collected per the screening standards of Yuan et al. (2022). From each block of 200 plants, 1 to 2 fruits per plant were pooled as one biological replicate, yielding three replicates per treatment.

For STw production, calyxes were removed, and fruits were homogenized under sterile conditions. Pectinase (0.03 g/L), potassium metabisulfite (0.06 g/L), and active dry yeast EC-1118 (0.20 g/L) were sequentially added, with sucrose to standardize initial sugar levels. Fermentation proceeded at 25 °C and was terminated when residual reducing sugar fell below 4 g/L. Each biological replicate was fermented independently in a separate fermenter. The crude wines were then filtered, supplemented with 0.03 g/L potassium metabisulfite, clarified at ambient temperature for 7 days, and matured at 15 °C for one month. Fermented samples were designated JDHW-1, JDHW-3, TSW-1, and TSW-3.

### 2.3 Physicochemical and color parameters

Total soluble solids (TSS, °Brix) and titratable acidity (TA) of fruits were determined using a PAL-BXI ACID F5 digital refractometer (Atago, Japan). Total soluble sugar was determined by the anthrone colorimetric assay (BC0035 kit) (http://www.shsolarbio.com/shsolarbio-Products-21681673/) and glucose and fructose were quantified according to the national standard method GB/T 5009.8-2016 of China. Results were normalized to fresh weight (mg/g FW). Basic physicochemical parameters of STw (total sugar, pH, total acid, volatile acid, dry extract, alcohol) were determined following GB/T 15038-2006. Wine color was measured by UV–Vis spectrophotometry (380–780 nm) in a 10 mm quartz cell, and CIELab parameters (L*, a*, b*, C*ab, *hab (°)*, ∆E*ab) were calculated according to Suberbiola et al. (1999).

### 2.4 Organic acids (OAs) analysis

OAs in fruits and ST_W_ were analyzed by HPLC (Bozan et al., 1997). Samples were mixed with 0.1% H_3_PO_4_, sonicated at 4 °C for 30 min, and centrifuged (5000 rpm, 5 min). After re-extraction with 0.1% H_3_PO_4_, combined supernatants were filtered (0.22 μm) and stored at 4 °C. Separation used a Waters XSelect HSS T3 column (250 × 4.6 mm, 3.5 μm) at 35 °C with 0.02 mol/L NH_4_H_2_PO_4_ (pH 2.7) at 0.9 mL/min, detected at 210 nm (10 μL injection). OAs were identified by retention time against a mixed standard of 12 acids prepared in 0.1% H_3_PO_4_. Method verification included limit of detection (LOD), limit of quantification (LOQ), precision and recovery. Results were expressed as mg/kg FW (fruits) and mg/L (wines).

### 2.5 Phenolics and antioxidant capacity analysis

#### 2.5.1 Total phenolic compounds and antioxidant capacity

Fresh fruits were lyophilized using a vacuum freeze dryer (SCIENTZ-12 N/A, China) and ground into powder. Briefly, 100 mg fruit powder was ultrasonically extracted with 1.5 mL of 80% aqueous methanol containing 1% HCl for 30 min, repeated three times, and centrifuged (10,000 rpm, 5 min, 4 °C), and the combined supernatants were retained. STw samples were analyzed directly without pre-extraction. Total phenolic content (TPC) and total flavonoid content (TFC) were determined by Folin-Ciocalteu and aluminum chloride methods (Wu et al., 2021), expressed as gallic acid equivalents (GAE) and rutin equivalents (RE), respectively. Total anthocyanin content (TAC) was measured by the pH differential method (Aramwit et al., 2010), and antioxidant capacity (AC) by DPPH, ABTS, and FRAP assays (Liang et al., 2022). All expressed as mM Trolox equivalents based on standard curves. Fruit values were reported as mg/100 g FW and wine values as mg/L.

#### 2.5.2 Monomeric phenols

Twenty-one monomeric phenols were quantified using ultra-high performance liquid chromatography coupled with tandem mass spectrometry (UPLC-MS/MS, Xevo TQ-S, Waters, USA) on an Agilent Poroshell EC-C18 column (2.1 × 150 mm, 2.7 μm) at 30 °C with a 1 μL injection. The mobile phase comprised 0.1% formic acid (A) and acetonitrile (B) at 0.3 mL/min under gradient elution: 0 min, 10% B; 1.5 min, 20% B; 4 min, 90% B; 8–10 min, 95% B; 10.1–12 min, 10% B. Analytes were detected by electrospray ionization (ESI) in positive/negative switching mode with multiple reaction monitoring (MRM), identified by matching retention times and fragment ions with authentic standards, and quantified via compound-specific calibration curves. Pooled QC samples were injected every 10 injections to monitor system stability, and LOD, LOQ, intra-day and inter-day precision were validated. Phenolic contents were expressed as mg/kg FW (fruits) and mg/L (wines).

### 2.6 Electronic tongue (*E*-tongue) analysis

Taste attributes of strawberry pulp and STw were measured using a SA402B E-tongue (Insent, Japan) equipped with six sensors for sourness, saltiness, umami, bitterness, astringency, and sweetness, also detecting aftertaste and richness. For fruits, 10.0 g pulp was homogenized with 150 mL distilled water, centrifuged (5000 rpm, 10 min, 4 °C), and the supernatant analyzed (Yu et al., 2022). For wines, 80 mL of clarified sample was equilibrated at 25 °C for 2 min before testing (Qian et al., 2023).

### 2.7 Volatile organic compounds (VOCs) analysis

VOCs were analyzed by GC-IMS (FlavourSpec®, G.A.S., Germany) following He et al. (2023). Briefly, 3.0 g fresh pulp or 1.5 mL STw was placed in a 20 mL vial with 10 μL of 20 μg/mL 4-methyl-2-pentanol as an internal standard, incubated at 45 °C for 15 min (500 rpm), and 500 μL headspace was injected (splitless, 85 °C). Separation was performed on an MXT®-WAX column (15 m × 0.53 mm × 1.0 μm, Restek, USA) at 60 °C with nitrogen carrier gas under programmed flow: 2.0 mL/min (0–2 min), 10.0 mL/min (2–10 min), 100.0 mL/min (10–40 min). Compounds were identified by drift time, retention index (C4–C9 ketones), and NIST 2020/GC-IMS library matching via VOCal® software. Semi-quantification was based on internal standard peak area normalization:

$$C_{i}=C_{\text{IS}}\times A_{i}/A_{\text{IS}}$$

where C_i_ and C_IS_ are the relative concentrations of the target compound and the internal standard, and A_i_ and A_IS_ are their peak areas. As no compound-specific calibration curves or response factors were established, all values are semi-quantitative internal standard equivalents (4-methyl-2-pentanol equivalents), not absolute concentrations. VOC contents were expressed as μg/g FW for fruits and μg/L for wines. Analytical reproducibility was assessed by long-term platform stability (RSD < 3%) and in-batch precision (RSD ≈ 0.57%, *n* = 45).

### 2.8 Statistical analysis

Data were analyzed using SPSS 22.0 and expressed as the mean ± standard deviation of three biological replicate determinations (SD). For all ANOVA and multivariate analyses, the biological replicate (independent plot, batch, or fermenter) served as the statistical unit. The effects of cultivar, HAR_T_ and their interaction were assessed by one-way and two-way ANOVA with Duncan's multiple-range test (*p* < 0.05). Pearson correlations among 12 biological samples were corrected with the Benjamini-Hochberg false discovery rate (FDR) (q < 0.05). Heatmaps were generated using TBtools (Chen et al., 2020), Mantel correlation heatmaps with ChiPlot (https://www.chiplot.online), and *E*-tongue radar fingerprints with OriginPro 2021. Principal component analysis (PCA) and orthogonal partial least squares discriminant analysis (OPLS-DA) were performed using SIMCA 14.1 on all 12 samples, with model validity assessed by R^2^ and Q^2^ using 200 permutation tests.

## 3 Results and discussion

### 3.1 Analysis of basic physicochemical properties and CIELab color parameters

HAR_T_ significantly influenced the basic physicochemical properties of both red and white strawberry fruits and their wines (Table 1). Two-way ANOVA showed significant cultivar × harvest interactions for TSS, fructose, glucose, and total soluble sugar (*p* < 0.01), and for SuAR and TA (*p* < 0.01 and *p* < 0.05) (Table S1). In the red JDH, harvest-dependent metabolic variations were associated with combined seasonal environmental factors. JDH-1 contained significantly higher TSS (12.00 ± 0.10°Brix), TA (0.72 ± 0.03%), sugar-acid ratio (SuAR, 16.68 ± 0.56), total soluble sugar (104.67 ± 3.14 mg/g FW), glucose (44.71 ± 2.55 mg/g FW), and fructose (46.90 ± 2.18 mg/g FW) (*p* < 0.05). The SuAR decreased by 24.3% from January to March, potentially influencing sweetness perception and consumer acceptability (Laaksonen et al., 2016). This trend was associated with the large diurnal temperature difference in January that may promoted carbon accumulation (Khammayom et al., 2022), while warmer March conditions were associated with enhanced organic acid respiration and reduced TA (Ruan et al., 2013). In contrast, white TS presented metabolically stable: TA, SuAR, and glucose did not differ between January and March (*p* > 0.05), consistent with spring-harvested strawberries (Ikegaya, 2023), indicating a stable sugar–acid balance favorable for winemaking.

**Table 1:** Basic physicochemical and color parameters of strawberries and wines.

Strawberry	TSS (°Brix)	TA (%)	SuAR	Fructose (mg/g FW)	Glucose (mg/g FW)	Total soluble sugar (mg/g FW) (mg/g FW)
JDH-1	12.00 ± 0.10a	0.72 ± 0.03b	16.68 ± 0.56a	46.90 ± 2.18a	44.71 ± 2.55a	104.67 ± 3.14a
JDH-3	8.03 ± 0.40d	0.64 ± 0.05c	12.63 ± 1.57b	32.69 ± 2.48c	29.56 ± 1.85c	80.59 ± 2.79c
TS-1	9.37 ± 0.06c	0.96 ± 0.05a	9.81 ± 0.46c	36.07 ± 1.78c	35.53 ± 1.95b	85.65 ± 4.58c
TS-3	10.03 ± 0.12b	1.02 ± 0.03a	9.84 ± 0.31c	40.33 ± 1.68b	38.28 ± 1.90b	94.87 ± 4.02b

**Wines**	**Alcohol (*v*/v)**	**Total acid (g/L)**	**Reducing** **sugar (g/L)**	**Dry extract (g/L)**	**Volatile acid (g/L)**	**pH**
JDHW-1	6.64 ± 0.08b	6.79 ± 0.08b	2.63 ± 0.11a	22.77 ± 0.51a	0.44 ± 0.02b	3.32 ± 0.01b
JDHW-3	6.82 ± 0.08a	5.18 ± 0.03c	2.71 ± 0.04a	22.40 ± 0.50a	0.47 ± 0.01a	3.39 ± 0.01a
TSW-1	6.63 ± 0.04bc	8.68 ± 0.15a	2.35 ± 0.04b	20.50 ± 0.17b	0.39 ± 0.01c	3.27 ± 0.02c
TSW-3	6.51 ± 0.04c	6.62 ± 0.53b	1.88 ± 0.06c	19.67 ± 0.40c	0.44 ± 0.02b	3.38 ± 0.01a
	L*	a*	b*	C*ab	***hab (°)***	ΔE*a
JDHW-1	80.63 ± 0.03d	23.26 ± 0.01a	33.45 ± 0.09b	40.75 ± 0.09a	55.19 ± 0.14c	0c
JDHW-3	82.05 ± 0.03c	13.77 ± 0.04b	34.16 ± 0.02a	36.83 ± 0.04b	68.04 ± 0.05b	9.62 ± 0.11a
TSW-1	97.06 ± 0.04a	1.10 ± 0.08c	9.63 ± 0.12d	9.70 ± 0.11d	83.51 ± 0.55a	0c
TSW-3	96.47 ± 0.04b	1.15 ± 0.22c	11.27 ± 0.68c	11.33 ± 0.65c	84.10 ± 1.52a	1.74 ± 0.47b

Note: Data are presented as mean ± standard deviation of three replicate determinations (SD). Different letters (a–d) in the same column indicate significant differences (*p <* 0.05) determined using the Duncan test.

All wines were fermented under uniform conditions (alcohol 6.51–6.82% v/v). Fresh fruit metabolic traits were inherited or remodeled during vinification depending on cultivar. Red wines retained the fruit acidity pattern (JDHW-1 > JDHW-3), consistent with TA variation in fresh samples (Fig. 1A). White wines displayed an opposite trend: despite higher TA in TS-3 fruits (1.02% vs. 0.96% in TS-1, 6.25% higher), TSW-3 exhibited lower acidity than TSW-1 (6.62 g/L vs. 8.68 g/L, 23.7% lower, *p* < 0.05), suggesting greater metabolic plasticity of the white cultivar, possibly linked to its higher malic acid content (Bokulich et al., 2014; Section 3.2). All wines were dry (reducing sugar <4 g/L), with volatile acid of 0.39–0.47 g/L, which may contribute to flavor complexity (Gamboa et al., 2019; Liu et al., 2018). JDH wines showed higher dry extract, possibly reflecting higher polyphenols and polysaccharides that may enhance taste intensity and persistence (Ascrizzi et al., 2022; Sáenz-Navajas et al., 2010).

**Fig. 1:**
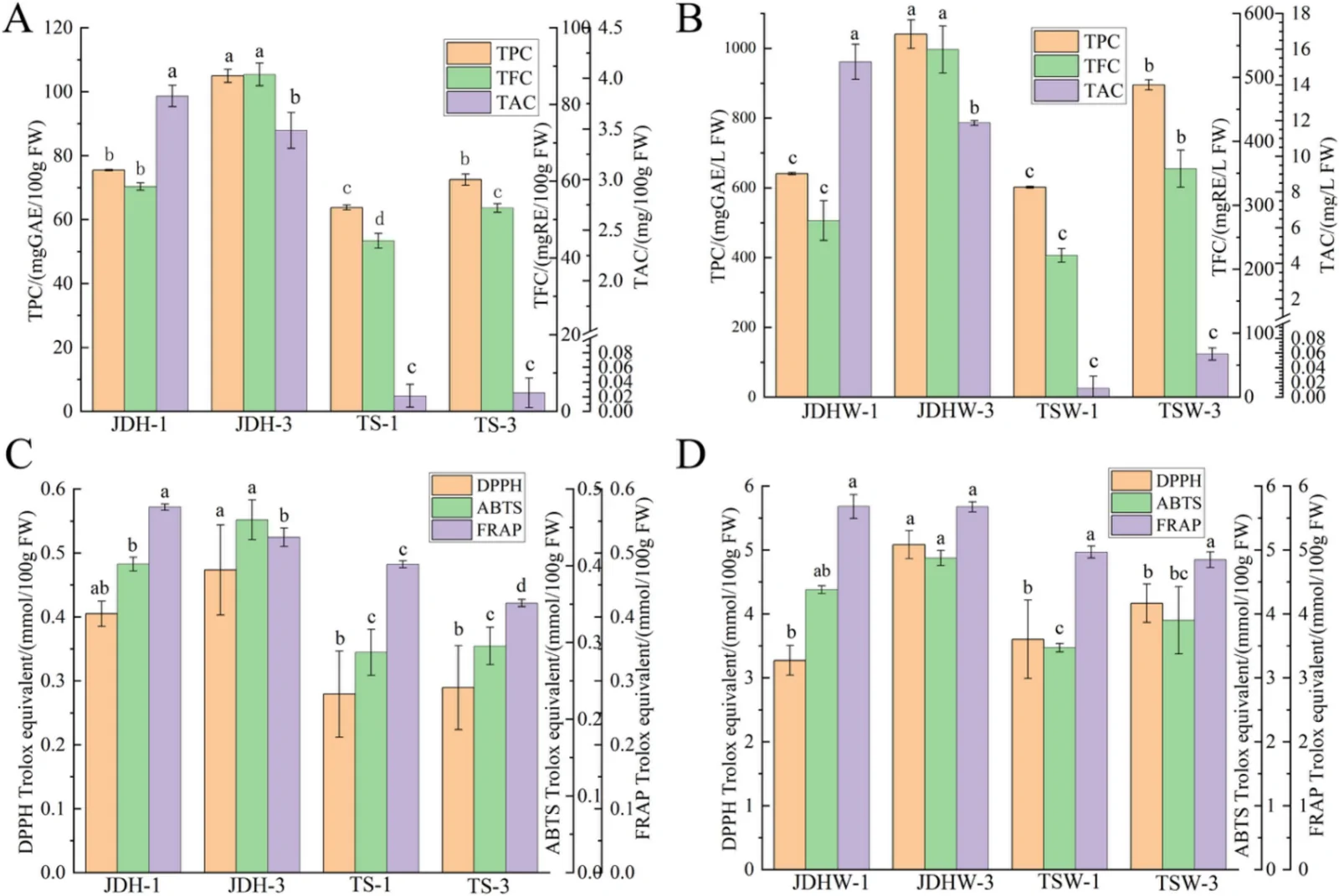
TPC, TFC, TAC and antioxidant capacities of strawberries and wines. Different lowercase letters above the error bars indicate significant differences among samples within the same parameter (*p* < 0.05, Duncan's test). Note that letters are not comparable across different Y-axes, as each parameter was analyzed independently.

CIELab analysis also showed cultivar-dependent harvest time effects. JDHW-3 had a higher L* value than JDHW-1 (increase of 1.42, *p* < 0.05), possibly because March conditions reduced anthocyanin stability during fermentation and aging (Shaked-Sachray et al., 2002). JDHW-3 also had lower a* and C*ab but higher b* and *hab (°)* (*p* < 0.05). The ΔE*ab of 9.62 exceeded the visual discrimination threshold of 3.0 (Ding et al., 2025), suggesting that HAR_T_ was associated with distinct color differences in red wines. In contrast, TSW-3 had a lower L* value than TSW-1 (*p* < 0.05), possibly due to higher non-anthocyanin phenols (Gao et al., 1997; Section 3.3), with higher b*, C*ab, and *hab (°)* (*p* < 0.05) enhancing yellow tone. The low ΔE*ab of 1.74 suggested negligible color variation. Significant C × HAR_T_ interactions were found for all CIELab parameters (*p* < 0.01 for *L*^*⁎*^, a*, Cab, and *hab (°)*; *p* < 0.05 for b*) and for alcohol, reducing sugar, and pH (*p* < 0.01; Table 2S), suggesting that harvest-associated color and compositional changes were modified by cultivar.

**Table 2:** Contents of 12 organic acids in strawberries and wines.

Compound	Strawberries (mg/kg FW)					Strawberry wines (mg/L)		
	JDH-1	JDH-3	TS-1	TS-3	JDHW-1	JDHW-3	TSW-1	TSW-3
Oxalic acid	–	–	–	–	309.70 ± 61.37 A	199.37 ± 7.53B	275.99 ± 29.77 A	–
Tartaric acid	4.35 ± 0.42d	6.32 ± 0.08c	13.98 ± 0.40a	9.01 ± 0.85b	–	–	–	–
Quinic acid	364.38 ± 21.41b	276.16 ± 18.33c	353.32 ± 24.84b	1001.18 ± 28.03a	2837.90 ± 695.48 A	1781.33 ± 347.63C	1828.52 ± 179.32 BCE	2572.94 ± 13.73AB
Malic acid	1070.03 ± 16.81c	702.68 ± 35.51d	1429.75 ± 10.99b	1653.58 ± 36.19a	3391.37 ± 631.10 A	1815.48 ± 60.44B	3544.68 ± 350.94 A	3788.369 ± 55.91 A
Malonic acid	–	–	–	–	–	–	–	–
Shikimic acid	1.38 ± 0.13c	1.45 ± 0.04c	2.70 ± 0.08b	3.66 ± 0.13a	0.13 ± 0.00C	3.46 ± 0.11B	–	12.04 ± 0.12 A
Lactic acid	–	–	–	–	729.20 ± 136.10 A	335.18 ± 8.15B	681.20 ± 79.52A	346.46 ± 4.80B
Acetic acid	–	–	–		2841.63 ± 622.63 A	1172.70 ± 113.84C	1837.39 ± 195.00B	757.50 ± 19.82C
Maleic acid	0.94 ± 0.00a	0.79 ± 0.02b	–	–	–	–	–	–
Citric acid	4936.85 ± 67.05c	4277.24 ± 135.63d	6989.81 ± 118.10a	6363.32 ± 150.57b	16,164.50 ± 2798.49B	11,696.50 ± 947.47C	21,689.38 ± 2419.10 A	14,085.61 ± 246.30 BCE
Fumaric acid	4.30 ± 0.11c	4.55 ± 0.21c	5.18 ± 0.23b	7.80 ± 0.31a	5.06 ± 0.85C	7.98 ± 0.83B	5.21 ± 1.14C	14.65 ± 0.60 A
Succinic acid	54.99 ± 1.33a	41.39 ± 5.74b	–	–	724.73 ± 75.51 A	362.28 ± 7.27C	511.44 ± 37.92B	329.36 ± 95.48C
**Total**	6509.88 ± 102.85b	5310.57 ± 288.21c	8794.73 ± 105.97a	9038.53 ± 215.19a	27,004.18 ± 4175.44AB	17,374.26 ± 1470.15C	30,373.79 ± 3277.91 A	21,906.95 ± 354.73 BCE

Note: “-” indicates that the item was not detected. Statistically significant differences (*p* < 0.05) in strawberry fruits between harvest seasons are indicated by different lowercase letters, while different uppercase letters denote significant differences in strawberry wines between the same seasons, according to Duncan's test.

### 3.2 Analysis of organic acid profiles

As shown in Table 2, citric acid (CA) dominated OAs in both cultivars, followed by malic, quinic, succinic, tartaric, and trace fumaric acid, consistent with the typical OA profile of Fragaria × ananassa (Gündüz & Özdemir, 2014). Succinic and maleic acids were undetectable in white TS fruit, while oxalic, lactic, acetic, and malonic acids were absent in all fresh samples. CA accounted for 70.40–80.54% of total OAs, ranging from 4277.24 ± 135.63 mg/kg FW (JDH-3) to 6989.81 ± 118.1 mg/kg FW (TS-1). Two-way ANOVA showed significant cultivar × HAR_T_ interactions for tartaric, quinic, shikimic, maleic, and fumaric acids (*p* < 0.001) and for succinic acid (*p* < 0.05) (Table S3). In red JDH, citric, malic and quinic acids declined significantly in JDH-3, leading to an 18.42% reduction in total OAs and lower TA. In contrast, white TS maintained stable acidity: although CA decreased in TS-3, compensatory accumulation of malic and quinic acids offset this loss. TS-3 exhibited 41.23% higher total OAs than JDH-3, highlighting the superior acid-buffering capacity of the white cultivar (Milosavljević et al., 2023). This cultivar-dependent buffering may explain the relatively stable malic and quinic acid levels in white fruit under warm harvest conditions (Ruan et al., 2013). Citric acid showed no significant C × HAR_T_ interaction in fruit; only additive main effects (harvest: *p* < 0.001, cultivar: *p* < 0.05) were observed, consistent across both cultivars (Table 2).

In wines, CA remained predominant, followed by malic, quinic, and acetic acids. Lactic, acetic, and oxalic acids were yeast-derived metabolites unique to wines (Yang et al., 2024), whereas succinic acid was detected only in white STw. Two-way ANOVA revealed significant C × HAR_T_ interactions for oxalic, quinic, shikimic, fumaric, succinic and citric acids (*p* < 0.001 for the former four; citric, *p* < 0.05, Table S3). Compared to JDHW-1, JDHW-3 showed a 35.66% reduction in total acid, with significant declines across all nine measurable acids. Despite stable TA in TS fruits, TSW-3 exhibited a 27.88% decrease in total acid relative to TSW-1, possibly related to the higher malic acid content in TS-3 enhancing yeast metabolic activity, as wine yeasts typically degrade 25–38% of malic acid (Redzepovic et al., 2003).

### 3.3 Analysis of phenolic compounds and antioxidant capacity

Cultivar and HAR_T_ significantly affected TPC, TFC, TAC, and antioxidant capacities (DPPH, ABTS, FRAP) in both fresh strawberries and wines (Fig. 1). March-harvested fruits of both cultivars had higher TPC and TFC, possibly associated with March conditions promoting phenolic accumulation (Balasooriya et al., 2019). DPPH and ABTS activities tracked phenolic levels, with JDH-3 strongest and TS-1 weakest. TAC and FRAP peaked in JDH-1, consistent with the darker color (low L*) of anthocyanin-rich red wines (Gutiérrez-Escobar et al., 2021), and were retained in wines (JDHW-1 highest, TSW-1 lowest). In JDH-3, March conditions may have been associated with enhanced non-anthocyanin phenolic synthesis and accelerated anthocyanin degradation (Balasooriya et al., 2019), resulting in lighter color (higher L*). In fresh fruits, two-way ANOVA showed that cultivar significantly affected all six parameters (*p* < 0.01); HAR_T_ affected TPC, TFC, TAC, and FRAP, but not DPPH or ABTS. Significant interactions occurred for TPC, TFC, and TAC only. In wines, cultivar significantly affected TPC, TFC, TAC, and ABTS (*p* < 0.01); HAR_T_ affected TPC, TFC, TAC, DPPH, and ABTS; interactions were significant for TPC, TAC, TFC, and DPPH, but not ABTS or FRAP.

Twenty-one phenolic monomers were quantified (Table 3). Flavan-3-ols (mainly catechin) dominated fresh fruits, followed by phenolic acids, aldehydes, and flavonols. Ellagic acid, the predominant phenolic acid, peaked in JDH-3 (10.55 ± 0.13 mg/kg FW). and JDHW-3 (1.70 ± 0.01 mg/L), possibly due to warm temperature activation of the phenylpropanoid pathway (Calzone et al., 2023; Urün et al., 2021). TS-1 accumulated exceptionally high catechin and vanillin (9.8-fold higher than JDH-1), associated with cold-induced acclimation and enhanced antioxidant defense (Xiang et al., 2020). In fresh fruits, 16 of 21 monomers showed significant cultivar × HAR_T_ interactions (*p* < 0.05; Table S4), including eight phenolic acids, two flavonols, epicatechin, catechin, and three aldehydes; syringic, ferulic, and sinapic acids responded additively, benzoic acid only by cultivar. In wines, 11 compounds, including protocatechuic, ferulic, p-coumaric, sinapic, ellagic and vanillic acids, kaempferol, rutin, epicatechin, catechin, and vanillin, showed significant interactions (*p* < 0.05; Table S4). JDHW-3 showed the highest catechin, p-hydroxybenzoic acid, ellagic acid, and ferulic acid levels, consistent with its strong antioxidant capacity. Other compounds were cultivar-independent: gallic and syringic acids and protocatechualdehyde/syringaldehyde were modulated mainly by harvest; caffeic and chlorogenic acids differed only among cultivars; p-hydroxybenzoic and benzoic acids responded to both factors without interaction; and o-coumaric acid showed no significant effect *(p* > 0.05). The catechin advantage weakened during vinification, yet vanillin and vanillic acid were well preserved, suggesting that white STw may be a promising candidate for fresh, low astringency products. Several compounds (syringic acid, o-coumaric acid, sinapic acid, protocatechualdehyde, syringaldehyde) were undetectable in all wines, suggesting complete degradation or biotransformation during vinification.

**Table 3:** Contents of 21 phenolic monomers in strawberries and wines.

Compound	Strawberries (mg/kg FW)				Strawberry wines (mg/L)			
	JDH-1	JDH-3	TS-1	TS-3	JDHW-1	JDHW-3	TSW-1	TSW-3
Phenolic acids								
Gallic acid	0.18 ± 0.00a	0.17 ± 0.00a	0.13 ± 0.00b	0.18 ± 0.01a	0.87 ± 0.02 A	0.50 ± 0.00B	0.85 ± 0.02 A	0.52 ± 0.01B
p-Hydroxybenzoic acid	0.14 ± 0.00b	0.16 ± 0.00a	0.11 ± 0.00d	0.13 ± 0.00c	1.71 ± 0.04B	1.79 ± 0.01 A	0.11 ± 0.01D	0.18 ± 0.01C
Vanillic acid	0.10 ± 0.00b	0.06 ± 0.00d	0.13 ± 0.00a	0.10 ± 0.00c	0.25 ± 0.01B	0.24 ± 0.00C	0.33 ± 0.00 A	0.17 ± 0.00D
Protocatechuic acid	0.01 ± 0.00d	0.02 ± 0.00a	0.01 ± 0.00c	0.02 ± 0.00b	0.10 ± 0.00B	0.16 ± 0.00 A	0.01 ± 0.00D	0.02 ± 0.00C
Syringic acid	0.02 ± 0.00b	0.01 ± 0.00d	0.02 ± 0.00a	0.01 ± 0.00c	0	0	0	0
Caffeic acid	0.08 ± 0.00b	0.14 ± 0.00a	0.03 ± 0.00d	0.04 ± 0.01c	0.21 ± 0.01 A	0.21 ± 0.00 A	0.10 ± 0.00B	0.10 ± 0.00B
Ferulic acid	0.06 ± 0.00d	0.07 ± 0.00c	0.08 ± 0.00b	0.11 ± 0.00a	0.01 ± 0.00C	0.07 ± 0.00 A	0.02 ± 0.00B	0.02 ± 0.00B
p-Coumaric acid	0.53 ± 0.00b	0.86 ± 0.01a	0.03 ± 0.00d	0.15 ± 0.01c	0.12 ± 0.00C	0.51 ± 0.00 A	0.14 ± 0.00B	0.08 ± 0.01D
o-Coumaric acid	0.01 ± 0.00	0.01 ± 0.00	0.01 ± 0.00	0.01 ± 0.00	0	0	0	0
Sinapic acid	0.02 ± 0.00a	0.01 ± 0.00b	0.01 ± 0.00b	0.01 ± 0.00b	0	0	0	0
Benzoic acid	1.23 ± 0.01b	1.19 ± 0.01b	1.80 ± 0.02a	1.82 ± 0.06a	0.10 ± 0.01 AB	0.09 ± 0.00B	0.11 ± 0.01 A	0.10 ± 0.01B
Chlorogenic acid	0.09 ± 0.00a	0.09 ± 0.00a	0.08 ± 0.01b	0.08 ± 0.00b	0.03 ± 0.00 A	0.03 ± 0.00 A	0.02 ± 0.00B	0.02 ± 0.00B
Ellagic acid	8.25 ± 0.04b	10.55 ± 0.13a	6.53 ± 0.07d	7.91 ± 0.11c	1.61 ± 0.05 A	1.70 ± 0.01 A	0.76 ± 0.21B	0.28 ± 0.02C
Total	10.71 ± 0.04b	13.35 ± 0.13a	8.99 ± 0.06d	10.56 ± 0.05c	5.02 ± 0.10B	5.31 ± 0.01 A	2.46 ± 0.18C	1.49 ± 0.06D

Flavonols								
Kaempferol	0.06 ± 0.00a	0.06 ± 0.00b	0.02 ± 0.00d	0.03 ± 0.00c	0.01 ± 0.00B	0.03 ± 0.00 A	0C	0C
Rutin	0.06 ± 0.00b	0.09 ± 0.00a	0.01 ± 0.00d	0.02 ± 0.00c	0.01 ± 0.00B	0.02 ± 0.00 A	0C	0C
Total	0.12 ± 0.00b	0.15 ± 0.00a	0.03 ± 0.00d	0.05 ± 0.00c	0.02 ± 0.00B	0.05 ± 0.00 A	0C	0C

Flavanols								
Epicatechin	0.33 ± 0.00d	0.51 ± 0.01c	0.56 ± 0.01b	0.81 ± 0.04a	0.05 ± 0.00C	0.10 ± 0.00B	0.09 ± 0.00B	0.10 ± 0.01 A
Catechin	269.68 ± 0.89d	346.89 ± 5.17b	382.08 ± 9.06a	311.27 ± 2.88c	37.21 ± 0.17C	63.83 ± 0.14 A	42.55 ± 2.10B	26.97 ± 0.88D
Total	270.00 ± 0.89d	347.39 ± 5.18b	382.64 ± 9.07a	312.08 ± 2.92c	37.26 ± 0.17C	63.92 ± 0.14 A	42.64 ± 2.10B	27.07 ± 0.89D
Phenolic aldehydes								
Protocatechualdehyde	0.01 ± 0.00bc	0.01 ± 0.00b	0.02 ± 0.00a	0.01 ± 0.01c	0	0	0	0
Syringaldehyde	0.04 ± 0.00b	0.04 ± 0.00a	0.04 ± 0.00b	0.04 ± 0.00b	0	0	0	0
Vanillin	0.04 ± 0.00d	0.06 ± 0.00c	0.39 ± 0.00a	0.20 ± 0.00b	0.01 ± 0.00C	0.01 ± 0.00C	0.04 ± 0.00 A	0.03 ± 0.00B
Total	0.09 ± 0.00d	0.11 ± 0.00c	0.45 ± 0.00a	0.25 ± 0.01b	0.01 ± 0.00C	0.01 ± 0.00C	0.04 ± 0.00 A	0.03 ± 0.00B

Simple phenols								
Catechol	–	–	–	–	–	–	–	–
Total	282.82 ± 0.89d	362.69 ± 5.26b	394.15 ± 9.04a	325.22 ± 2.93c	42.41 ± 0.24B	69.40 ± 0.14 A	45.28 ± 2.27B	28.71 ± 0.97C

Note: Different lowercase letters indicate significant differences among strawberry samples across harvest dates, while different uppercase letters indicate significant differences among wine samples across harvest dates, according to Duncan's test (*p* < 0.05).

### 3.4 Analysis of taste profiles via *E*-tongue

E-tongue taste-related sensor responses (Table S2) were plotted as a radar chart (Fig. 2A). The overall radar contour shapes were similar among samples, but specific taste attributes differed notably between red and white cultivars and across harvest dates. For fresh fruits, most responses overlapped except for sourness and sweetness. JDH-3 and TS-1 showed lower sweetness responses than JDH-1 and TS-3, respectively, consistent with their SuAR. Among wines, TSW-1 exhibited the highest sourness response, followed by JDHW-1, JDHW-3, and TSW-3, basically consistent with OA and TA contents.

**Fig. 2:**
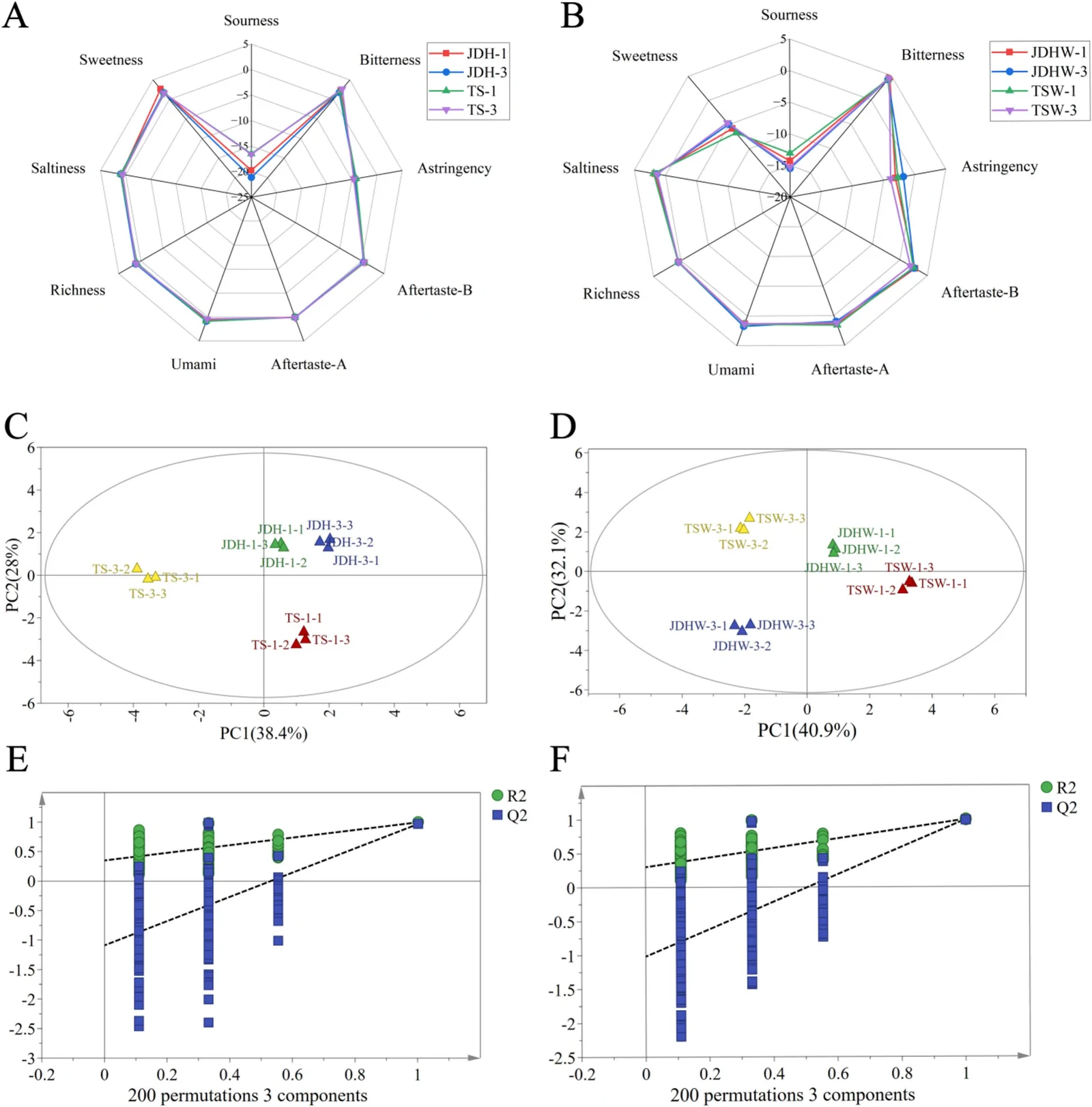
E-tongue analysis for different strawberries and wines. Radar chart (A and B). OPLS-DA models based on the response values of E-tongue sensors (C and D). Permutation tests of the OPLS-DA model (E and F).

OPLS-DA was further applied to the E-tongue response (Fig. 2B), which clearly distinguished all four fruit types and all four wines. Seven-fold cross-validation yielded satisfactory parameters (fruit model: R^2^X = 0.968, R^2^Y = 0.989, Q^2^ = 0.965; wine model: R^2^X = 0.984, R^2^Y = 0.998, Q^2^ = 0.990) with R^2^ and Q^2^ values well above 0.5 (Yun et al., 2021). As shown in Fig. 2C, after 200 permutation tests (Fig. 2C), the intercept of the Q^2^ intercept on the vertical axis was below zero and all permuted R^2^ and Q^2^ values were lower than their original counterparts, confirming no overfitting. No overlap was observed among different fruit or wine samples, highlighting distinct taste fingerprints associated with cultivar and HAR_T_. Among the nine taste attributes, four (sweetness, aftertaste-B, sourness and aftertaste-A) had variable importance in projection (VIP) > 1 in the fruit model, and aftertaste-B, astringency and bitterness were the most influential (VIP > 1) in the wine model, indicating their strong contribution to sample discrimination. These trends appear consistent with the compositional data described above. The sweetness response of fresh fruits followed SuAR rather than individual sugar concentrations, supporting the view that sweet taste perception depends on the balance between sugars and acids (Hao et al., 2025; Ikegaya, 2023). The sourness response of wines broadly followed total acid and organic acid contents, with TSW-1 showing the highest sourness response, consistent with its highest total acid. The distinct taste fingerprints of white and red wines may also reflect differences in phenolic composition: TS wines retained more vanillin and vanillic acid, whereas JDH wines were richer in ellagic acid and catechin. Together, these findings indicate that *E*-tongue taste fingerprints provide an instrumental basis for the cultivar- and HAR_T_-dependent differentiation of strawberry fruits and their fermented wines.

### 3.5 Analysis of volatile profiles

Two-dimensional differential spectral comparisons (Fig. 3A and B) revealed that VOC signals were mainly distributed within retention times of 200–800 s and drift times of 1.0–2.0 ms. Subtracting March spectra from the January baseline revealed distinct red/blue regions in JDH fruits (200–800 s), indicating significant VOC fluctuations. The corresponding wines showed dense two-color clusters, suggesting extensive metabolic recombination during fermentation. TS also varied across harvest dates, but less pronounced than JDH, confirming the central role of genetic background in VOC responses to HAR_T_ (Mavromatis et al., 2021).

**Fig. 3:**
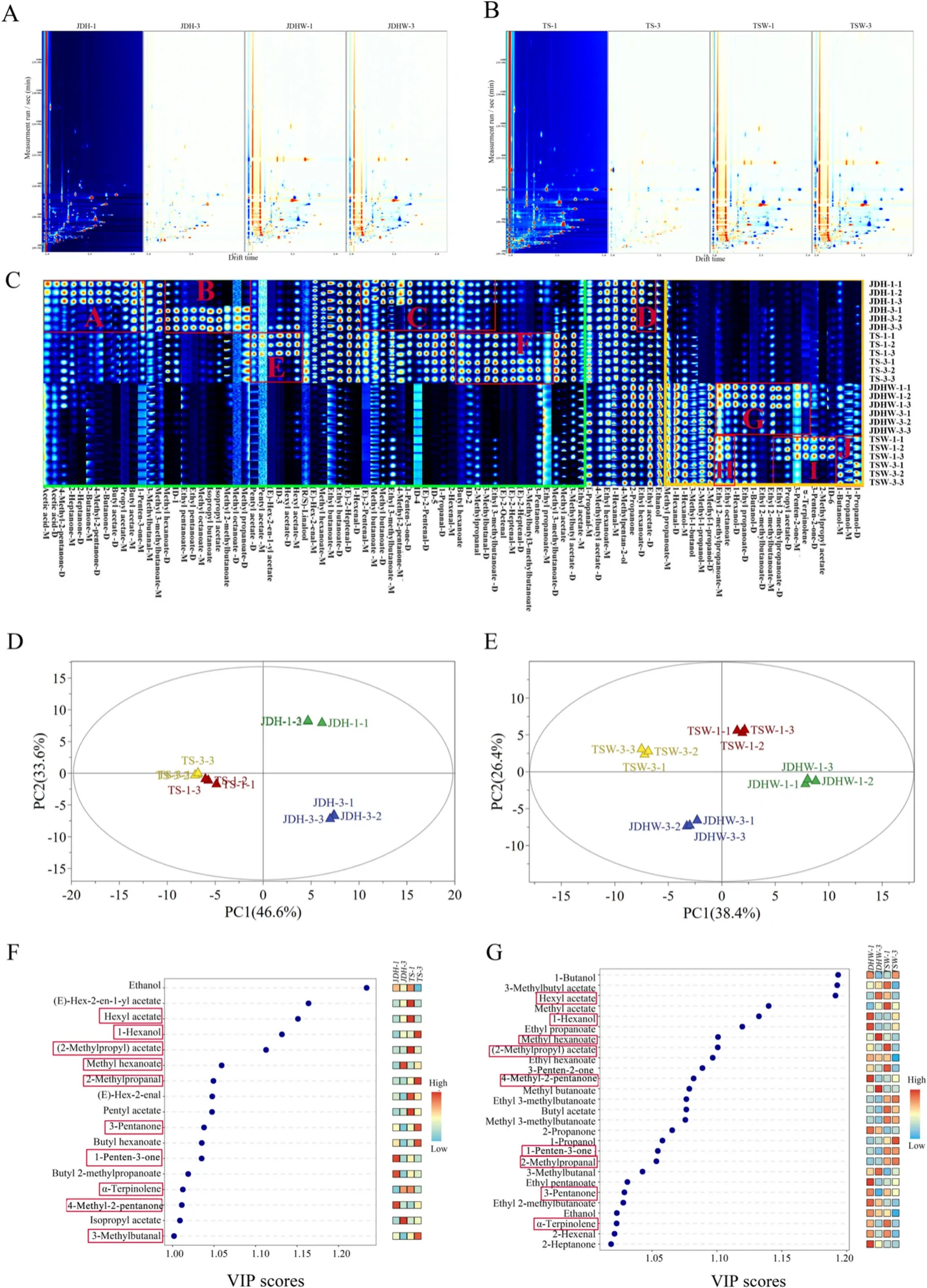
VOCs in different strawberries and their wines. (A and B) 2D-topographic comparison plots of VOCs. (C) Fingerprint chromatograms of VOCs. (D and E) OPLS-DA analysis of VOCs. (F and G) Heat map of VOCs (VIP > 1, Kruskal-Wallis H test (*p* < 0.05)).

GC-IMS identified 95 peaks corresponding to 88 VOCs, including 46 esters, 16 aldehydes, 12 ketones, 10 alcohols, 2 acids, and 2 terpenes (Table S3). Most appeared as monomers and dimers, consistent with concentration-dependent aggregation (Arce et al., 2014), and esters, alcohols, and aldehydes dominated in both fruits and wines (Table S4). The total VOC relative concentrations were higher in January-harvested fruits for both cultivars: JDH-1 (8.80 ± 0.35 μg/g FW) > JDH-3 (8.28 ± 0.03 μg/g FW), TS-1 (9.36 ± 0.35 μg/g FW) > TS-3 (9.22 ± 0.10 μg/g FW) (*p* < 0.05). The same trend was observed in wines: JDHW-1 (20,953.95 ± 173.69 μg/L) > JDHW-3 (17,346.16 ± 255.32 μg/L), TSW-1 (20,207.13 ± 178.70 μg/L) > TSW-3 (16,319.74 ± 126.17 μg/L) (*p* < 0.05). In JDH, the March harvest significantly increased relative ester content while decreasing aldehydes/ketones, acids, and terpenes (*p* < 0.05). In TS, no significant changes were observed in esters, alcohols, aldehydes/ketones, or acids across harvest dates, though terpenes declined similarly (*p* < 0.05).

Fingerprint and heatmap analyses (Fig. 3C) resolved ten VOC regions (A–J). Regions A, C, and D (JDH-1) were enriched with diverse esters, aldehydes, ketones, and acids, primarily ethyl acetate, while region B (JDH-3) was dominated by seven esters, such as methyl octanoate, methyl hexanoate, and ethyl hexanoate. Region E (TS-1) featured three fruity esters (hexyl acetate, (E)-2-hexenyl acetate, pentyl acetate), associated with strawberry fruity characteristics (Sheng et al., 2021), whereas region F (TS-3) contained esters, aldehydes, and ketones, including ethyl propionate, butyl hexanoate, (E)-2-heptenal, and 3-pentanone. In wines, region G (JDHW-1) was characterized by ethyl octanoate, ethyl propionate, 3-penten-2-one, 1-butanol, 1-hexanol, and α-terpinene; regions H and I (TSW-1) by α-terpinene, propyl acetate, ethyl 2-methylpropanoate, ethyl octanoate, and 3-penten-2-one; and region J (TSW-3) by elevated 11-propanol and 1-butanol.

Among characteristic compounds, ethyl acetate, ethyl hexanoate, and ethyl butyrate are well-established markers in strawberry and its products (Gaborieau et al., 2018; Wu et al., 2019; Yang et al., 2009). Their levels were significantly reduced in JDH-3 relative to JDH-1, indicating that the typical flavor accumulation in red strawberries is affected by HAR_T._ No such difference occurred in TS, suggesting a cultivar-dependent response. January-harvested JDH-1 also accumulated higher levels of aldehydes associated with fresh and green attributes, especially (E)-2-hexenal, a key freshness-related compound (Lindhorst & Steinhaus, 2016). This was markedly reduced in March samples (Du et al., 2022). JDH-1 also accumulated 2-methylpropanal and 3-methylbutanal, contributing to the green flavor basis of strawberries (Aharoni et al., 2004). Lower aldehyde content in March-harvested JDH was associated with changes in the lipoxygenase (LOX) pathway, whereas altered ester composition suggested HAR_T_- dependent shifts in alcohol acyltransferase (AAT) substrate specificity or carboxymethyltransferase (CMT) activation (Fang et al., 2024).

In wines, α-terpinolene was significantly elevated in both JDHW-1 and TSW-1, suggesting that January harvest promotes terpene glycosidic precursors accumulation, subsequently hydrolyzed by yeast β-glucosidases (Lv et al., 2024). Terpenes have extremely low odor thresholds and may enhance wine aroma complexity (Wu et al., 2019). Ketones, such as 3-penten-2-one, were also higher in wines from January-harvested fruits, potentially contributing floral and fruity notes (Pittari et al., 2021). Increased 1-propanol and 1-butanol in TSW-3 (region J) may be associated with higher precursor levels in March-harvested fruits or altered yeast amino acid metabolism (Hazelwood et al., 2008; Styger et al., 2011). Alcohols generally impart stronger ethanol-like and grassy notes (Styger et al., 2011); excessive levels may mask delicate fruit aromas, a potential quality disadvantage for white wines from March-harvested fruits.

OPLS-DA models for fruits and wines (Fig. 3D-E) successfully discriminated all sample groups. The fruit model showed R^2^X = 0.925, R^2^Y = 0.996, Q^2^ = 0.987; the wine model yielded R^2^X = 0.864, R^2^Y = 0.997, Q^2^ = 0.988. A 200-permutation test verified model reliability without overfitting (Fig. S1). Based on VIP > 1 and the Kruskal-Wallis H test (*p* < 0.05), 17 marker compounds (8 esters, 3 aldehydes, 2 alcohols, 3 ketones, and 1 terpene) were identified in fruits, and 27 markers (13 esters, 3 aldehydes, 4 alcohols, 6 ketones, and 1 terpene) in wines (Fig. 3F-G). Two-way ANOVA of the 17 fruit markers revealed significant cultivar × HAR_T_ interactions for all 17 (*p* < 0.05), with most main effects also significant (*p* < 0.001). Exceptions included α-terpinolene (significant interaction only) and ethanol (significant HAR_T_ effect and interaction only). In wines, all but two of the 27 markers showed significant interactions (*p* < 0.05); the cultivar main effect was significant for all 27, whereas the HAR_T_ main effect was non-significant only for pent-1-en-3-one. 3-Methylbutanal behaved additively (interaction *p* = 0.901). Collectively, genetic background and HAR_T_ acted as interdependent determinants of VOC responses, with the interaction prevailing in both fruits and wines.

### 3.6 Correlation analysis of sugars, organic acids, phenolics, and taste attributes

Pearson correlation and Mantel tests were performed on metabolites (34 in fresh fruits, 32 in wines) to explore associations between chemical components and *E*-tongue taste signals. Pearson correlations were globally corrected using Benjamini–Hochberg FDR (q < 0.05), whereas Mantel tests were corrected within each taste attribute (m = 34 for fruits, m = 32 for wines; q < 0.05).

In fresh fruits (Fig. 4A), phenolics and organic acids showed specific coupling: rutin and chlorogenic acid were negatively correlated with malic acid and CA (q < 0.001), while vanillin was positively correlated with tartaric acid (q < 0.001). Kaempferol and sinapic acid correlated with maleic acid and succinic acid (q < 0.001), caffeic acid with p-coumaric acid (q < 0.001, *r* = 0.996), and ellagic acid with caffeic acid (q < 0.001, *r* = 0.953), reflecting shared biosynthetic precursors (e.g., pentose phosphate and shikimate pathways) (Kajdžanoska et al., 2010). 268 of the 561 pairwise comparisons remained significant after FDR correction. Mantel tests (Fig. 4A) showed that *E*-tongue sweetness response was associated with SuAR (q < 0.05, *r* = 0.90), and the sourness response with TA (q < 0.05, *r* = 0.94) and pH (q < 0.05, *r* = 0.91), suggesting combined sugar-acid effects (Hao et al., 2025; Ikegaya, 2023). Aftertaste-B and aftertaste-A responses were linked to several phenolic acids (e.g., syringic, protocatechuic, ellagic, and ferulic acids), whereas umami and bitterness responses showed no significant associations.

**Fig. 4:**
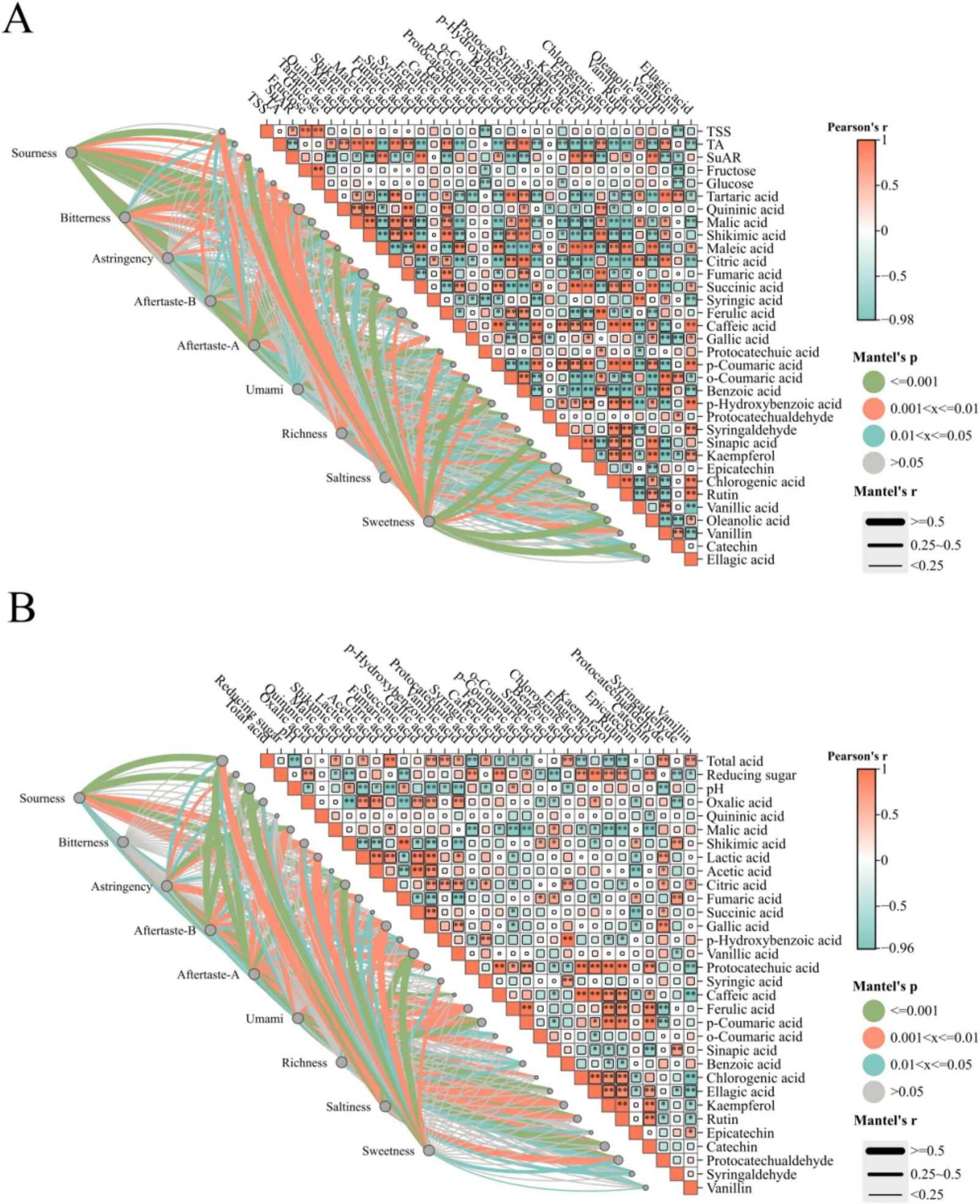
Mantel correlation analysis between E-tongue sensor responses and compounds (monosaccharides, organic acids, and phenolics) in fresh strawberries (A) and their wines (B). In the upper triangular segment, each square represents the Pearson's rank correlation coefficient between compounds; color denotes its value (green, negative; orange, positive). The links between the molecular profiles and E-tongue sensors indicate Mantel correlations, with line color and thickness representing the r and *p* values, respectively. * *p* < 0.05; ** *p* < 0.01. (For interpretation of the references to color in this figure legend, the reader is referred to the web version of this article.)

In wines (Fig. 4B), the network was partially reshaped (107 of 496 pairwise comparisons significant). Ellagic acid correlated with caffeic acid (q < 0.001, *r* = 0.71), catechin with p-coumaric acid (q < 0.001, *r* = 0.95), and syringaldehyde negatively with oxalic acid (q < 0.05, *r* = −0.75) but positively with shikimic (q < 0.05, *r* = 0.77) and fumaric acid (q < 0.05, *r* = 0.78). Mantel tests showed *E*-tongue sourness response was associated with pH (q < 0.05, *r* = 0.91), total acid (q < 0.05, *r* = 0.75) and citric acid (q < 0.05, *r* = 0.73); the astringency response with catechin and p-coumaric acid (q < 0.001, *r* = 0.94; q < 0.001, *r* = 0.85), consistent with the mechanism by which flavan-3-ols and phenolic acids interact with salivary proteins (Mercurio & Smith, 2008); and aftertaste-B response with reducing sugar, shikimic, oxalic, and ellagic acids (*r* = 0.74–0.90). E-tongue bitterness and richness responses showed no compound surviving q < 0.05 after FDR correction. Umami, saltiness, and sweetness responses were also associated with several organic acids and phenolics, though with lower r values. Correlation does not establish causation, and experimental validation is needed.

### 3.7 Correlation analysis of sugars, OAs, phenolics and key VOCs

The unique flavor of fruit wine originates from the complex interactions between volatile and non-volatile components (Lian et al., 2025). Pearson correlation analysis was performed to explore associations of sugars, OAs and phenolics with VOCs in fruits and wines.

In fruits (Fig. 5A), the network among organic acids, phenolics, and VOCs was extensively established (151 of 527 pairwise comparisons remained significant after BH–FDR). Fructose and glucose showed positive correlation with isobutyl butyrate (q < 0.001, *r* = 0.84 and 0.88, respectively), consistent with their role in supplying pyruvate and acetyl-CoA for ester biosynthesis (Gong et al., 2020). Sugars also positively correlated with 1-penten-3-one and methyl isobutyl ketone (q < 0.05), suggesting that sufficient sugar metabolism may be associated with LOX-pathway aldehyde/ketone formation (Boss et al., 2014). Malic acid was positively correlated with isovaleraldehyde (q < 0.001, *r* = 0.96), isobutyraldehyde (q < 0.001, *r* = 0.87), tartaric and malic acids with (E)-2-hexenal (Tartaric: q < 0.001, *r* = 0.90; Malic: q < 0.05, *r* = 0.75), and quinic, shikimic and fumaric acids with the branched-chain aldehydes (Quinic-isovaleraldehyde, Shikimic, Fumaric) (All q < 0.001), suggesting that OAs may supply carbon skeletons for branched-chain amino acid catabolism toward fresh green aldehydes (Boss et al., 2014; Du et al., 2022). Among phenolics, catechin correlated with (E)-2-hexen-1-yl acetate (q < 0.001, *r* = 0.83) and hexyl acetate (q < 0.001, *r* = 0.83), and o-coumaric acid with pentyl acetate (q < 0.001, *r* = 0.92), potentially through hydrophobic interactions and π–π stacking affecting VOC release (Pozo-Bayón & Reineccius, 2009). In contrast, ellagic and caffeic acids generally showed negative correlations with VOCs, possibly due to a salting-out effect that enhances VOC solubility and limits volatilization (Milenković et al., 2017).

**Fig. 5:**
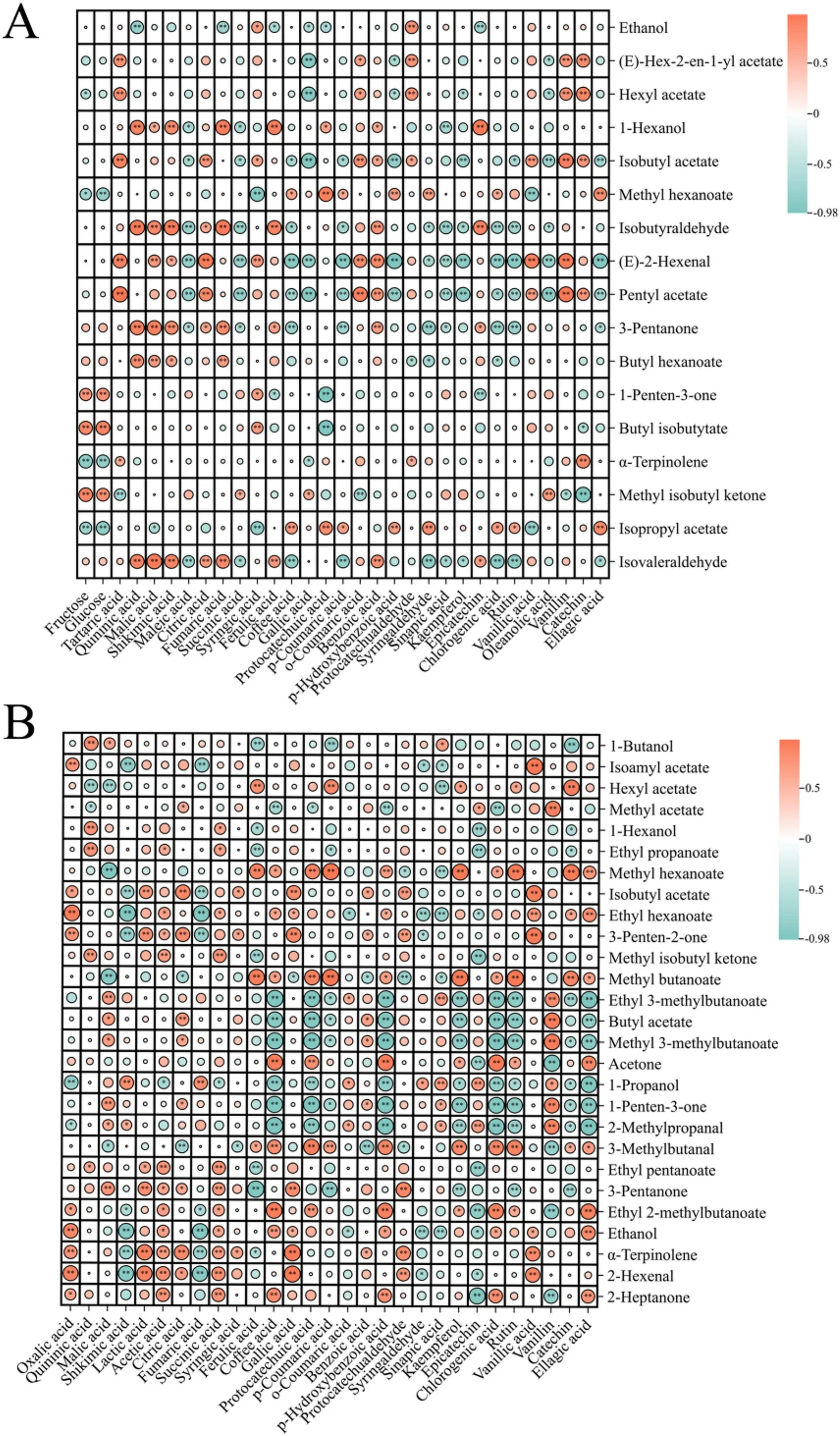
Pearson correlation analysis of compounds (monosaccharides, organic acids, and phenolics) and VOCs in different strawberries (A) and their wines (B). Red and blue cells indicate positive and negative correlations, respectively. * indicates a significant difference with *p* < 0.05 and ** indicates a significant difference with *p* < 0.01. (For interpretation of the references to color in this figure legend, the reader is referred to the web version of this article.)

In wines (Fig. 5B), the phenolic/organic-acid–VOC network was extensively reshaped (164 of 783 comparisons significant). p-Coumaric acid correlated with hexyl acetate (q < 0.001, *r* = 0.96), vanillic acid with isobutyl acetate (q < 0.001, *r* = 0.96), and catechin with hexyl acetate (q < 0.001, *r* = 0.94), whereas ellagic and kaempferol were negatively correlated with ethyl 3-methylbutanoate (ellagic: q < 0.001, *r* = −0.95; kaempferol: q < 0.001, *r* = −0.96). Notably, ellagic acid was simultaneously positively correlated with methyl hexanoate (q < 0.001, *r* = 0.91), and shikimic acid was negatively correlated with pent-3-en-2-one and (E)-2-hexenal (q < 0.001; *r* = −0.91 for both). These contrasting associations indicate that phenolic compounds exert bidirectional effects on VOC release: the same phenolic can promote or suppress different volatiles depending on its chemical structure, concentration, and the surrounding matrix. Possible mechanisms include hydrophobic interactions and π–π stacking that enhance VOC retention, versus salting-out effects or enzyme inhibition that reduce specific volatiles (Angerosa et al., 2000; Yang et al., 2024). Organic acids, by contrast, may primarily influence green-aldehyde formation through precursor supply and modulation of enzyme activities.

## 4 Conclusion

This study shows that HAR_T_ and cultivar jointly shape strawberry fruit and wine quality in a cultivar-dependent manner, with significant cultivar × harvest interactions for most parameters. A central finding is that in white strawberry (TS) wine, color remained stable despite harvest-driven reshaping of phenolics, volatiles, and *E*-tongue taste profiles, unlike red (JDH) wines, which showed pronounced color variation. This contrast is consistent with distinct metabolic responses red JDH exhibited organic acid degradation and color deviation under March harvest, alongside enhanced phenolic accumulation, whereas white TS displayed strong metabolic buffering, with compensatory malic and quinic acid accumulation maintaining stable total acidity. This cultivar-specific metabolic memory partially persisted through fermentation: catechin decreased in TS, while vanillin and vanillic acid were selectively retained, and January harvest enriched terpenes, aldehydes, and ketones in both cultivars. E-tongue responses discriminated samples, with the sourness response associated with total acid and pH, and astringency response with catechin and p-coumaric acid. These associations remain correlative and await targeted validation.

These findings support differentiated utilization: January-harvested TS is suited for retaining catechin and vanillin, whereas March-harvested JDH yields wines rich in ellagic acid and esters. Within the scope of this study (two harvest dates, a single year, and no human sensory evaluation), these compositional findings are offered as preliminary evidence. The rational pairing of cultivar and harvest period may nevertheless provide a practical lever for steering acid balance, phenolic accumulation, volatile composition, color, and E-tongue taste profiles, offering tentative guidance for germplasm utilization and fermentation management. Multi-environment and multi-season trials with sensory validation will be valuable to confirm and extend these findings.

## CRediT authorship contribution statement

**Hongmin Liang:** Writing – original draft, Formal analysis, Conceptualization. **Cong Wang:** Resources, Investigation, Data curation. **Xiaoyu Yu:** Visualization, Validation, Software. **Yanxia Zhao:** Writing – review & editing, Funding acquisition, Conceptualization. **Jiabo Sun:** Supervision, Project administration, Funding acquisition.

## Funding

Agricultural Science & Technology Innovation Project of Shandong Academy of Agricultural Sciences (CXGC2026B30, CXGC2026H22).

## Declaration of competing interest

The authors declare that they have no known competing financial interests or personal relationships that could have appeared to influence the work reported in this paper.

## Data Availability

Data will be made available on request.
