# Supplementary material for: Harvest time differentially shapes flavor compounds in red and white strawberry cultivars (Fragaria × ananassa ‘Jiandehong’ and ‘Tianshi’) and their wines via cultivar-specific metabolic responses
Source: Food Chem X. 2026 Oct 13;40:104622. doi: 10.1016/j.fochx.2026.104622 (PMC13718864; doi:10.1016/j.fochx.2026.104622)
Supplement: Supplementary file 1 [file mmc1.docx]

**Supplementary Data**

**Table S1. Environmental conditions in the greenhouse during the 7-day period preceding each sampling day**

| **7-day period** | **Temp**  **Mean (°C)** | **Temp Max**  **(_◦_C)** | **Temp Min**  **(◦C)** | **RH**  **Mean (%)** | **RH**  **Max (%)**  **(%)** | **RH**  **Min (%)**  **(%)** | **Rain**  **(mm)** |
| --- | --- | --- | --- | --- | --- | --- | --- |
| 8–14 January | 12.11 ± 0.89 | 26.61 ± 2.04 | 5.53 ± 1.17 | 62.10 ± 1.57 | 84.37 ± 1.07 | 15.47 ± 0.98 | 0 |
| 20–26 March | 18.89 ± 3.28 | 35.31 ± 1.21 | 7.40 ± 1.14 | 47.60 ± 7.31 | 73.13 ± 5.37 | 13.74 ± 1.43 | 0 |

Note: Environmental data were recorded by an S21A-2100 temperature and relative humidity sensor. For each 7-day period (including the sampling day), daily mean, maximum, and minimum values were first calculated; the table reports the mean ± SD of these daily values (n = 7). Temp max mean and Temp min mean represent the average of daily maximum and minimum temperatures, respectively. Rain indicates total precipitation during the 7-day period; no rainfall occurred in either period.

**Table S2. Significance values of two-way ANOVA for the effects of cultivar (C), HAR_T_ and their interaction (C × HAR_T_) on physicochemical properties and color parameters of different strawberries and wines**

| **NO**. | **Variables** | **Strawberry fruits** | | | **Variables** | **Strawberry wines** | | |
| --- | --- | --- | --- | --- | --- | --- | --- | --- |
|  |  | **C** | **HAR_T_** | **C × HAR_T_** |  | **C** | **HAR_T_** | **C × HAR_T_** |
| 1 | TSS | * | ** | ** | Alcohol | ** | ns | ** |
| 2 | SuAR | ** | ns | ** | Total acid | ** | ** | ns |
| 3 | TA | ** | ** | ** | Reducing sugar | ** | ** | ** |
| 4 | SuAR | ns | ** | ** | Dry extract | ** | * | ns |
| 5 | Fructose | ns | ** | ** | Volatile acid | ** | ** | ns |
| 6 | Glucose | ns | ** | ** | pH | ** | ** | ** |
| 7 | Total soluble sugar | ** | ** | ** | *L** | ** | ** | ** |
| 8 | TPC | ** | ** | ** | *a** | ** | ** | ** |
| 9 | TFC | ** | * | * | *b** | ** | ** | * |
| 10 | TAC | ** | ns | ns | *C*ab* | ** | ** | ** |
| 11 | DPPH | ** | ns | ns | *hab (°)* | ** | ** | ** |
| 12 | ABTS | ** | ** | ns | TPC | ** | ** | ** |
| 13 | FRAP |  |  |  | TFC | ** | ** | ** |
| 14 |  |  |  |  | TAC | ** | ** | ** |
| 15 |  |  |  |  | DPPH | ns | ** | * |
| 16 |  |  |  |  | ABTS | ** | * | ns |
| 17 |  |  |  |  | FRAP | ns | ns | ns |

Note: Data were analyzed by two-way ANOVA with cultivar (C) and harvest time (HAR_T_) as fixed factors. Significance levels: ns, not significant (*p* ≥ 0.05); *, *p* < 0.05; **, *p* < 0.01. Each treatment was analyzed with three biological replicates.

**Table S3. Significance values of two-way ANOVA for the effects of cultivar (C), HAR_T_ and their interaction (C × HAR_T_) on organic acid profiles of different strawberries and wines**

| **NO**. | **Organic acids** | **Strawberry fruits** | | | **Strawberry wines** | | |
| --- | --- | --- | --- | --- | --- | --- | --- |
|  |  | **C** | **HAR_T_** | **C × HAR_T_** | **C** | **HAR_T_** | **C × HAR_T_** |
| 1 | Oxalic acid | - | - | - | * | ** | ** |
| 2 | Tartaric acid | ****** | ***** | ****** | **-** | **-** | **-** |
| 3 | Quinic acid | ****** | ****** | ****** | n**s** | n**s** | ****** |
| 4 | Malic acid | ****** | ***** | ****** | ****** | ****** | ***** |
| 5 | Malonic acid | **-** | **-** | **-** | **-** | **-** |  |
| 6 | Shikimic acid | ****** | ****** | ****** | ****** | ****** | ****** |
| 7 | Lactic acid | **-** | **-** | **-** | n**s** | ****** | n**s** |
| 8 | Acetic acid | **-** | **-** | **-** | **-** | **-** |  |
| 9 | Maleic acid | ****** | ****** | ****** | **-** | **-** | **-** |
| 10 | Citric acid | ***** | ****** | n**s** | ****** | ****** | ***** |
| 11 | Fumaric acid | ****** | ****** | ****** | ****** | ****** | ****** |
| 12 | Succinic acid | ****** | ***** | ***** | ****** | ****** | ***** |

Note: Data were analyzed by two-way ANOVA with cultivar (C) and harvest time (HAR_T_) as fixed factors. Significance levels: ns, not significant (*p* ≥ 0.05); *, *p* < 0.05; **, *p* < 0.01. “-” indicates the compound was not detected or not applicable. Each treatment was analyzed with three biological replicates.

**Table S4. Significance values of two-way ANOVA for the effects of cultivar (C), HAR_T_ and their interaction (C × HAR_T_) on phenolic monomer profiles of different strawberries and wines**

| **NO**. | **Phenolic monomers** | **Strawberry fruits** | | | **Strawberry wines** | | |
| --- | --- | --- | --- | --- | --- | --- | --- |
|  |  | **C** | **HAR_T_** | **C × HAR_T_** | **C** | **HAR_T_** | **C × HAR_T_** |
| 1 | Gallic acid | ****** | ****** | ****** | n**s** | ****** | n**s** |
| 2 | p-Hydroxybenzoic acid | ****** | ****** | ***** | ****** | ****** | n**s** |
| 3 | Vanillic acid | ****** | ****** | ****** | n**s** | ****** | ****** |
| 4 | Protocatechuic acid | ****** | ****** | ****** | ****** | ****** | ****** |
| 5 | Syringic acid | ****** | ****** | n**s** | n**s** | ***** | n**s** |
| 6 | Caffeic acid | ****** | ****** | ****** | ****** | n**s** | n**s** |
| 7 | Ferulic acid | ****** | ****** | n**s** | ****** | ****** | ****** |
| 8 | p-Coumaric acid | ****** | ****** | ****** | ****** | ****** | ****** |
| 9 | o-Coumaric acid | ****** | n**s** | ****** | n**s** | n**s** | n**s** |
| 10 | Sinapic acid | ****** | ***** | n**s** | ****** | n**s** | ****** |
| 11 | Benzoic acid | ****** | n**s** | n**s** | ***** | ***** | n**s** |
| 12 | Chlorogenic acid | ****** | ****** | ****** | ****** | n**s** | n**s** |
| 13 | Ellagic acid | ****** | ****** | ****** | ****** | ***** | ****** |
| 14 | Kaempferol | ****** | ****** | ****** | ****** | ****** | ****** |
| 15 | Rutin | ****** | ****** | ****** | ****** | ****** | ****** |
| 16 | Epicatechin | ****** | ****** | ***** | ****** | ****** | ****** |
| 17 | Catechin | ****** | n**s** | ****** | ****** | ****** | ****** |
| 18 | Protocatechualdehyde | n**s** | ****** | ****** | n**s** | ****** | n**s** |
| 19 | Syringaldehyde | ****** | ***** | ****** | n**s** | ***** | n**s** |
| 20 | Vanillin | ****** | ****** | ****** | ****** | n**s** | ****** |
| 21 | Catechol | **-** | **-** | **-** | **-** | **-** | **-** |

Note: Data were analyzed by two-way ANOVA with cultivar (C) and harvest time (HAR_T_) as fixed factors. Significance levels: ns, not significant (*p* ≥ 0.05); *, *p* < 0.05; **, *p* < 0.01. “-” indicates the compound was not detected or not applicable. Each treatment was analyzed with three biological replicates.

**Table S5. E-tongue response values of different strawberries and wines**

| **Sensor** | **Sensitive** | **Strawberry fruits** | | | | | **Strawberry wines** | | | | |
| --- | --- | --- | --- | --- | --- | --- | --- | --- | --- | --- | --- |
|  |  | **JDH-1** | **JDH-3** | **TS-1** | **TS-3** | **VIP** | **JDHW-1** | **JDHW-3** | **TSW-1** | **TSW-3** | **VIP** |
| CA0 | Sourness | -19.75 ± 0.38b | -21.13 ± 0.35c | -16.50 ± 0.48a | -16.61 ± 0.60a | 1.02 | -14.29 ± 0.17b | -15.46 ± 1.16c | -13.07 ± 0.17a | -15.23 ± 0.35c | 0.966 |
| CO0 | Bitterness | 2.69 ± 0.18a | 1.80 ± 0.09b | 2.16 ± 0.39b | 2.72 ± 0.27a | 0.95 | 4.64 ± 0.12a | 4.19 ± 0.25b | 4.19 ± 0.08b | 4.52 ± 0.05a | 1.02 |
| AE1 | Astringency | -4.41 ± 0.12b | -4.30 ± 0.09b | -4.13 ± 0.09a | -4.62 ± 0.10c | 0.95 | -3.08 ± 0.23c | -1.81 ± 0.12a | -2.70 ± 0.15b | -3.85 ± 0.25d | 1.01 |
| - | Aftertaste-B | 0.63 ± 0.03a | 0.38 ± 0.02c | 0.62 ± 0.03a | 0.48 ± 0.04b | 1.06 | 2.75 ± 0.02a | 2.62 ± 0.04b | 2.46 ± 0.01c | 1.89 ± 0.03d | 1.11 |
| - | Aftertaste-A | 0.03 ± 0.01c | 0.05 ± 0.01b | 0.06 ± 0.01b | 0.11 ± 0.02a | 1.01 | 1.35 ± 0.12b | 0.97 ± 0.07d | 1.59 ± 0.02a | 1.21 ± 0.03c | 0.97 |
| AAE | Umami | 0.54 ± 0.09b | 0.86 ± 0.13a | 0.88 ± 0.20a | 0.39 ± 0.10c | 0.91 | 1.50 ± 0.51a | 1.53 ± 0.36a | 1.39 ± 0.08a | 1.66 ± 0.47b | 0.74 |
| - | Richness | 1.09 ± 0.08a | 1.20 ± 0.13a | 0.85 ± 0.10b | 1.07 ± 0.11a | 0.98 | 0.41 ± 0.02b | 0.47 ± 0.03a | 0.49 ± 0.04a | 0.43 ± 0.04ab | 0.97 |
| CT0 | Saltiness | 0.84 ± 0.14a | 1.01 ± 0.12a | 1.04 ± 0.20a | 0.47 ± 0.18b | 0.91 | 1.71 ± 0.04b | 0.41 ± 0.02d | 1.90 ± 0.05a | 1.31 ± 0.10c | 0.97 |
| GL1 | Sweetness | 2.66 ± 0.06a | 1.75 ± 0.11b | 1.52 ± 0.02c | 1.64 ± 0.03bc | 1.06 | -5.82 ± 0.51b | -4.95 ± 0.26a | -6.70 ± 0.41c | -4.57 ± 0.19a | 0.94 |

Note: The data are presented as mean ± standard deviation of three replications (SD). Different letters (a–d) in the same row indicate significant differences (*p* < 0.05) based on the Duncan test.

**Table S6. Characterization of VOCs based on GC-IMS**

| **NO**. | **Compound** | **CA** | **Formula** | **MW** | **RI** | **Rt [s]** | **Dt** | **Odor characteristics** |
| --- | --- | --- | --- | --- | --- | --- | --- | --- |
|  | Esters (46) |  |  |  |  |  |  |  |
| **Esters (46)** | | | | | | | | |
| 1 | Ethyl octanoate | C106321 | C10H20O2 | 172.3 | 1,441.8 | 969.969 | 1.48751 | fruity, pineapple, apple, brandy |
| 2 | Methyl octanoate-M | C111115 | C9H18O2 | 158.2 | 1,395.9 | 838.568 | 1.44242 | fruity, orange, wine, wax |
| 3 | Methyl octanoate-D | C111115 | C9H18O2 | 158.2 | 1,394.9 | 836.016 | 1.95333 | fruity, orange, wine, wax |
| 4 | (E)-Hex-2-en-1-yl acetate | C2497189 | C8H14O2 | 142.2 | 1,342.1 | 707.167 | 1.8923 | sweet, apple peel, banana peel |
| 5 | Hexyl acetate-M | C142927 | C8H16O2 | 144.2 | 1,281 | 587.821 | 1.39836 | fruity, green, apple, banana, sweet |
| 6 | Hexyl acetate-D | C142927 | C8H16O2 | 144.2 | 1,281.9 | 589.027 | 1.9292 | fruity, green, apple, banana, sweet |
| 7 | Ethyl hexanoate-M | C123660 | C8H16O2 | 144.2 | 1,236.7 | 526.991 | 1.34701 | pineapple, fruity, wine |
| 8 | Ethyl hexanoate-D | C123660 | C8H16O2 | 144.2 | 1,235.8 | 525.802 | 1.83001 | pineapple, fruity, wine |
| 9 | Methyl hexanoate-M | C106707 | C7H14O2 | 130.2 | 1,186.5 | 465.572 | 1.29948 | pineapple, apricot, fruity |
| 10 | Methyl hexanoate-D | C106707 | C7H14O2 | 130.2 | 1,186.5 | 465.572 | 1.71459 | pineapple, apricot, fruity |
| 11 | Pentyl acetate-M | C628637 | C7H14O2 | 130.2 | 1,172.2 | 448.93 | 1.32664 | bananas, apples, pears |
| 12 | Pentyl acetate-D | C628637 | C7H14O2 | 130.2 | 1,172.8 | 449.722 | 1.79122 | bananas, apples, pears |
| 13 | 3-Methylbutyl acetate-M | C123922 | C7H14O2 | 130.2 | 1,123.6 | 396.097 | 1.31843 | sweet, banana, fruity |
| 14 | 3-Methylbutyl acetate-D | C123922 | C7H14O2 | 130.2 | 1,123.4 | 395.888 | 1.7782 | sweet, banana, fruity |
| 15 | Ethyl pentanoate-M | C539822 | C7H14O2 | 130.2 | 1,143.2 | 416.625 | 1.28945 | apple, pineapple, green |
| 16 | Ethyl pentanoate-D | C539822 | C7H14O2 | 130.2 | 1,143.2 | 416.634 | 1.69637 | apple, pineapple, green |
| 17 | Butyl acetate-M | C123864 | C6H12O2 | 116.2 | 1,077.7 | 353.383 | 1.24834 | fruity |
| 18 | Butyl acetate-D | C123864 | C6H12O2 | 116.2 | 1,077.1 | 352.921 | 1.64679 | fruity |
| 19 | Ethyl 3-methylbutanoate-M | C108645 | C7H14O2 | 130.2 | 1,070.3 | 347.842 | 1.26424 | apple, banana, sour and sweet |
| 20 | Ethyl 3-methylbutanoate-D | C108645 | C7H14O2 | 130.2 | 1,069.4 | 347.15 | 1.68234 | apple, banana, sour and sweet |
| 21 | Ethyl 2-methylbutanoate-M | C7452791 | C7H14O2 | 130.2 | 1,053.9 | 335.839 | 1.25208 | apple |
| 22 | Ethyl 2-methylbutanoate-D | C7452791 | C7H14O2 | 130.2 | 1,052.9 | 335.146 | 1.67859 | apple |
| 23 | Ethyl butanoate-M | C105544 | C6H12O2 | 116.2 | 1,039.5 | 325.682 | 1.21373 | pineapple, fruity, ester, whiskey |
| 24 | Ethyl butanoate-D | C105544 | C6H12O2 | 116.2 | 1,039.5 | 325.682 | 1.58693 | pineapple, fruity, ester, whiskey |
| 25 | Methyl 3-methylbutanoate-M | C556241 | C6H12O2 | 116.2 | 1,022.9 | 314.37 | 1.21373 | strong apple, pineapple |
| 26 | Methyl 3-methylbutanoate-D | C556241 | C6H12O2 | 116.2 | 1,020.7 | 312.87 | 1.55059 | strong apple, pineapple |
| 27 | Methyl butanoate-M | C623427 | C5H10O2 | 102.1 | 993.8 | 295.441 | 1.15574 | apple, sweet banana, pineapple |
| 28 | Methyl butanoate-D | C623427 | C5H10O2 | 102.1 | 991.6 | 294.056 | 1.45224 | apple, sweet banana, pineapple |
| 29 | Propyl acetate-D | C109604 | C5H10O2 | 102.1 | 978.3 | 286.207 | 1.49994 | fruity, pear |
| 30 | Propyl acetate-M | C109604 | C5H10O2 | 102.1 | 979.1 | 286.669 | 1.17538 | fruity, pear |
| 31 | Ethyl propanoate-D | C105373 | C5H10O2 | 102.1 | 961.8 | 276.974 | 1.47095 | grape, pineapple, fruity, rum |
| 32 | Ethyl propanoate-M | C105373 | C5H10O2 | 102.1 | 961.4 | 276.764 | 1.15456 | grape, pineapple, fruity, rum |
| 33 | Isopropyl acetate | C108214 | C5H10O2 | 102.1 | 910 | 249.965 | 1.49994 | fruity |
| 34 | Ethyl acetate-D | C141786 | C4H8O2 | 88.1 | 900.6 | 245.348 | 1.35216 | fresh, fruity, sweet, grassy |
| 35 | Ethyl acetate-M | C141786 | C4H8O2 | 88.1 | 901.4 | 245.775 | 1.10047 | fresh, fruity, sweet, grassy |
| 36 | Methyl acetate | C79209 | C3H6O2 | 74.1 | 866.7 | 229.42 | 1.20625 | ethereal |
| 37 | Methyl propanoate-D | C554121 | C4H8O2 | 88.1 | 919.2 | 254.582 | 1.3372 | Fruit, Rum |
| 38 | Methyl propanoate-M | C554121 | C4H8O2 | 88.1 | 918.3 | 254.132 | 1.09896 | Fruit, Rum |
| 39 | Ethyl 2-methylpropanoate-D | C97621 | C6H12O2 | 116.2 | 968.5 | 280.667 | 1.58412 | sweet, fruity, alcoholic, rummy |
| 40 | Ethyl 2-methylpropanoate-M | C97621 | C6H12O2 | 116.2 | 966.8 | 279.744 | 1.21375 | sweet, fruity, alcoholic, rummy |
| 41 | Methyl 2-methylbutanoate | C868575 | C6H12O2 | 116.2 | 1,012.8 | 307.676 | 1.55794 | apple |
| 42 | Isopropyl butanoate | C638119 | C7H14O2 | 130.2 | 1,043.2 | 328.271 | 1.70987 | Fruit, Pungent |
| 43 | Butyl hexanoate | C626824 | C10H20O2 | 172.3 | 1,396.8 | 840.937 | 1.48532 | fruity |
| 44 | 3-Methylbutyl 3-methylbutanoate | C659701 | C10H20O2 | 172.3 | 1,299.6 | 617.865 | 1.46688 | apple, banana |
| 45 | Butyl 2-methylpropanoate | C97870 | C8H16O2 | 144.2 | 1,151.7 | 425.829 | 1.32633 | fruity, sweet |
| 46 | (2-Methylpropyl) acetate | C110190 | C6H12O2 | 116.2 | 1,014.5 | 308.751 | 1.63593 | fruity, raw pear and raspberry |
| **Alcohols (10)** | | | | | | | | |
| 47 | 1-Hexanol-M | C111273 | C6H14O | 102.2 | 1,363 | 755.388 | 1.33687 | fresh, fruity, wine, sweet, green |
| 48 | 1-Hexanol-D | C111273 | C6H14O | 102.2 | 1,364 | 757.799 | 1.66275 | fresh, fruity, wine, sweet, green |
| 49 | 3-Methyl-1-butanol | C123513 | C5H12O | 88.1 | 1,207 | 489.743 | 1.25196 | whiskey, banana, fruity |
| 50 | 1-Butanol-M | C71363 | C4H10O | 74.1 | 1,143.1 | 416.437 | 1.18698 | wine |
| 51 | 1-Butanol-D | C71363 | C4H10O | 74.1 | 1,143.1 | 416.437 | 1.39259 | wine |
| 52 | Ethanol | C64175 | C2H6O | 46.1 | 940.3 | 265.432 | 1.13423 | aromaticity |
| 53 | 2-Methyl-1-propanol-M | C78831 | C4H10O | 74.1 | 1,092.6 | 365.593 | 1.1811 | fresh, alcoholic, leather |
| 54 | 2-Methyl-1-propanol-D | C78831 | C4H10O | 74.1 | 1,094 | 366.982 | 1.38382 | fresh, alcoholic, leather |
| 55 | 1-Propanol-M | C71238 | C3H8O | 60.1 | 1,039.1 | 325.417 | 1.11607 | alcohol, pungent |
| 56 | 1-Propanol-D | C71238 | C3H8O | 60.1 | 1,038.8 | 325.178 | 1.26516 | alcohol, pungent |
| **Aldehydes (16)** | | | | | | | | |
| 57 | (E)-2-Hexenal-M | C6728263 | C6H10O | 98.1 | 1,219.9 | 505.593 | 1.18795 | green, banana, fat |
| 58 | (E)-2-Hexenal-D | C6728263 | C6H10O | 98.1 | 1,220.3 | 505.989 | 1.53905 | green, banana, fat |
| 59 | (E)-2-Pentenal-M | C1576870 | C5H8O | 84.1 | 1,133.1 | 405.911 | 1.11337 | potatoes, peas |
| 60 | (E)-2-Pentenal-D | C1576870 | C5H8O | 84.1 | 1,132.9 | 405.716 | 1.37542 | potatoes, peas |
| 61 | 1-Hexanal-M | C66251 | C6H12O | 100.2 | 1,089.9 | 363.078 | 1.2633 | fresh, green, fat, fruity |
| 62 | 1-Hexanal-D | C66251 | C6H12O | 100.2 | 1,090.2 | 363.309 | 1.58225 | fresh, green, fat, fruity |
| 63 | 1-Propanal-M | C123386 | C3H6O | 58.1 | 844.4 | 219.494 | 1.07436 | pungent, green grassy |
| 64 | 1-Propanal-D | C123386 | C3H6O | 58.1 | 840.6 | 217.874 | 1.15348 | pungent, green grassy |
| 65 | 2-Methylpropanal | C78842 | C4H8O | 72.1 | 824.3 | 210.952 | 1.09775 | banana, melon, slightly nutty |
| 66 | (E)-2-Octenal | C2548870 | C8H14O | 126.2 | 1,430.1 | 934.545 | 1.3465 | fresh cucumber, fatty, green herbal, banana, green leaf |
| 67 | (E)-2-Heptenal-M | C18829555 | C7H12O | 112.2 | 1,327.6 | 675.273 | 1.2663 | spicy, green vegetables, fresh, fatty |
| 68 | (E)-2-Heptenal-D | C18829555 | C7H12O | 112.2 | 1,326.8 | 673.633 | 1.69512 | spicy, green vegetables, fresh, fatty |
| 69 | 2-Hexenal-M | C505577 | C6H10O | 98.1 | 1,202.8 | 484.648 | 1.18743 | sweet almonds, fruity, green leaves, apples, plums, vegetables |
| 70 | 2-Hexenal-D | C505577 | C6H10O | 98.1 | 1,203.7 | 485.786 | 1.53312 | sweet almonds, fruity, green leaves, apples, plums, vegetables |
| 71 | 3-Methylbutanal-M | C590863 | C5H10O | 86.1 | 927.4 | 258.734 | 1.17628 | chocolate, fat |
| 72 | 3-Methylbutanal-D | C590863 | C5H10O | 86.1 | 928.6 | 259.369 | 1.42069 | chocolate, fat |
| **Ketones (12)** | | | | | | | | |
| 73 | 3-Pentanone | C96220 | C5H10O | 86.1 | 982.4 | 288.516 | 1.36993 | ethereal |
| 74 | 2-Propanone | C67641 | C3H6O | 58.1 | 855.9 | 224.572 | 1.12674 | fresh, apple, pear |
| 75 | 4-Methyl-2-pentanone-D | C108101 | C6H12O | 100.2 | 1,011.8 | 306.983 | 1.50275 | ketone |
| 76 | 4-Methyl-2-pentanone-M | C108101 | C6H12O | 100.2 | 1,010.7 | 306.291 | 1.18661 | ketone |
| 77 | 2-Heptanone-M | C110430 | C7H14O | 114.2 | 1,179 | 456.946 | 1.27203 | pear, banana, fruity, slight medicinal fragrance |
| 78 | 2-Heptanone-D | C110430 | C7H14O | 114.2 | 1,179.7 | 457.705 | 1.65532 | pear, banana, fruity, slight medicinal fragrance |
| 79 | 1-Penten-3-one-M | C1629589 | C5H8O | 84.1 | 1,027.9 | 317.757 | 1.07911 | strong pungent odors |
| 80 | 1-Penten-3-one-D | C1629589 | C5H8O | 84.1 | 1,027.5 | 317.44 | 1.32646 | strong pungent odors |
| 81 | 2-Butanone-M | C78933 | C4H8O | 72.1 | 915.5 | 252.718 | 1.06421 | fruity, camphor |
| 82 | 2-Butanone-D | C78933 | C4H8O | 72.1 | 915 | 252.461 | 1.25914 | fruity, camphor |
| 83 | 3-Penten-2-one-M | C625332 | C5H8O | 84.1 | 1,129.5 | 402.078 | 1.08122 | fruity, turns into spicy during storage |
| 84 | 3-Penten-2-one-D | C625332 | C5H8O | 84.1 | 1,128.8 | 401.363 | 1.35228 | fruity, turns into spicy during storage |
| **Acids (2)** | | | | | | | | |
| 85 | Acetic acid-M | C64197 | C2H4O2 | 60.1 | 1,478.1 | 1,088.11 | 1.05608 | spicy |
| 86 | Acetic acid-D | C64197 | C2H4O2 | 60.1 | 1,478.2 | 1,088.704 | 1.15815 | spicy |
| **Terpenes (2)** | | | | | | | | |
| 87 | (RS)-Linalool | C78706 | C10H18O | 154.3 | 1,581.7 | 1,511.245 | 1.23747 | citrus, rose, woody, blueberry |
| 88 | α-Terpinolene | C586629 | C10H16 | 136.2 | 1,283 | 590.718 | 1.3078 | fresh woody sweet pine citrus |

MW: Molecular weight. RI: Retention index calculated.

Rt: Retention time in the capillary GC column. Dt: The drift time in the drift tube.

M: Monomer; D: Dimer.

Odor characteristics are obtained from: [https://www.thegoodscentscompany.com](https://www.thegoodscentscompany.com/) ([Guan et al., 2025](https://www.sciencedirect.com/science/article/pii/S0963996925011287?via=ihub#bb0100))

**Table S7. The relative content of VOCs based on GC-IMS**

| **NO.** | **Compound** | **VOCs (**μg/g **FW)** | | | | **VOCs (**μg**/L)** | | | |
| --- | --- | --- | --- | --- | --- | --- | --- | --- | --- |
|  |  | **JDH-1** | **JDH-3** | **TS-1** | **TS-3** | **JDHW-1** | **JDHW-3** | **TSW-1** | **TSW-3** |
| **Esters (46)** | | | | | | | | | |
| 1 | Ethyl octanoate | 0.02 ± 0.00a | 0.02 ± 0.00b | 0.01 ± 0.00c | 0.01 ± 0.00c | 455.06 ± 13.09A | 225.56 ± 4.80C | 394.39 ± 0.36B | 230.78 ± 4.11C |
| 2 | Methyl octanoate-M | 0.03 ± 0.0b | 0.11 ± 0.00a | 0.03 ± 0.00b | 0.03 ± 0.00b | 69.07 ± 3.59A | 49.44 ± 0.13B | 65.20 ± 11.06A | 53.17 ± 3.88B |
| 3 | Methyl octanoate-D | 0.01 ± 0.00a | 0.01 ± 0.00a | 0.01 ± 0.00a | 0.01 ± 0.00a | 17.76 ± 1.80B | 17.60 ± 2.15B | 18.02 ± 1.28B | 20.37 ± 3.86A |
| 4 | (E)-Hex-2-en-1-yl acetate | 0.02 ± 0.00b | 0.02 ± 0.01b | 0.04 ± 0.00a | 0.01 ± 0.00b | 50.11 ± 5.60B | 55.25 ± 19.12A | 46.10 ± 3.58C | 46.86 ± 4.11C |
| 5 | Hexyl acetate-M | 0.14 ± 0.00c | 0.20 ± 0.05b | 0.26 ± 0.01a | 0.13 ± 0.01c | 56.56 ± 6.90C | 208.80 ± 6.85A | 160.59 ± 5.81B | 36.53 ± 1.86D |
| 6 | Hexyl acetate-D | 0.04 ± 0.00b | 0.12 ± 0.08b | 0.33 ± 0.02a | 0.05 ± 0.00b | 32.55 ± 2.29C | 69.28 ± 2.92A | 56.64 ± 2.27B | 31.81 ± 1.06C |
| 7 | Ethyl hexanoate-M | 0.19 ± 0.01a | 0.14 ± 0.00b | 0.12 ± 0.01c | 0.13 ± 0.00c | 428.56 ± 13.55B | 467.69 ± 6.63A | 424.20 ± 7.92B | 354.21 ± 7.20C |
| 8 | Ethyl hexanoate-D | 0.35 ± 0.01a | 0.15 ± 0.01c | 0.17 ± 0.01c | 0.20 ± 0.01b | 1,068.78 ± 3.85A | 886.36 ± 9.99C | 973.60 ± 6.63B | 332.17 ± 11.75D |
| 9 | Methyl hexanoate-M | 0.18 ± 0.01b | 0.21 ± 0.00a | 0.17 ± 0.01c | 0.16 ± 0.00c | 177.74 ± 13.62B | 236.25 ± 3.98A | 160.29 ± 5.42C | 137.35 ± 4.01D |
| 10 | Methyl hexanoate-D | 0.41 ± 0.02c | 0.80 ± 0.01a | 0.41 ± 0.03c | 0.54 ± 0.01b | 91.14 ± 9.46B | 136.93 ± 0.54A | 94.72 ± 4.47B | 75.10 ± 4.69C |
| 11 | Pentyl acetate-M | 0.01 ± 0.00b | 0.01 ± 0.00b | 0.02 ± 0.00a | 0.01 ± 0.00b | 35.89 ± 0.88A | 26.62 ± 1.30B | 25.69 ± 2.67BC | 22.05 ± 2.94C |
| 12 | Pentyl acetate-D | 0.00 ± 0.00b | 0.00 ± 0.00b | 0.01 ± 0.00a | 0.00 ± 0.00b | 13.26 ± 1.41A | 12.37 ± 1.35B | 13.60 ± 1.46A | 12.06 ± 1.47B |
| 13 | 3-Methylbutyl acetate-M | 0.08 ± 0.01b | 0.09 ± 0.00a | 0.08 ± 0.00b | 0.07 ± 0.00c | 205.47 ± 8.77C | 249.99 ± 2.46A | 237.25 ± 4.64B | 138.94 ± 2.21D |
| 14 | 3-Methylbutyl acetate-D | 0.10 ± 0.00d | 0.13 ± 0.00c | 0.50 ± 0.03a | 0.47 ± 0.00b | 486.76 ± 2.41C | 539.73 ± 6.57B | 922.99 ± 6.72A | 92.67 ± 6.36D |
| 15 | Ethyl pentanoate-M | 0.01 ± 0.00b | 0.04 ± 0.00a | 0.01 ± 0.00b | 0.01 ± 0.00b | 43.88 ± 4.46A | 20.59 ± 1.07C | 25.71 ± 0.48B | 27.56 ± 1.41B |
| 16 | Ethyl pentanoate-D | 0.00 ± 0.00b | 0.02 ± 0.00a | 0.00 ± 0.00b | 0.00 ± 0.00b | 8.99 ± 0.40A | 7.78 ± 1.58B | 7.90 ± 0.60B | 7.94 ± 1.14B |
| 17 | Butyl acetate-M | 0.04 ± 0.00a | 0.03 ± 0.00b | 0.01 ± 0.00c | 0.01 ± 0.00c | 11.24 ± 1.24B | 9.10 ± 0.17C | 18.08 ± 1.35A | 12.72 ± 0.81B |
| 18 | Butyl acetate-D | 0.10 ± 0.00a | 0.03 ± 0.00d | 0.06 ± 0.00c | 0.07 ± 0.00b | 16.21 ± 2.82C | 11.51 ± 0.52D | 32.04 ± 1.73A | 26.27 ± 1.94B |
| 19 | Ethyl 3-methylbutanoate-M | 0.13 ± 0.01a | 0.06 ± 0.00d | 0.13 ± 0.01a | 0.11 ± 0.00b | 104.17 ± 2.80C | 45.73 ± 1.81D | 201.27 ± 2.55B | 231.29 ± 2.13A |
| 20 | Ethyl 3-methylbutanoate-D | 0.19 ± 0.01c | 0.02 ± 0.00d | 0.38 ± 0.02b | 0.46 ± 0.00a | 44.29 ± 2.67C | 20.14 ± 0.39D | 275.43 ± 1.72B | 316.62 ± 9.99A |
| 21 | Ethyl 2-methylbutanoate-M | 0.01 ± 0.0b | 0.02 ± 0.00a | 0.02 ± 0.00a | 0.2 ± 0.00a | 123.04 ± 4.55A | 90.76 ± 1.53B | 49.65 ± 3.44C | 28.77 ± 2.64D |
| 22 | Ethyl 2-methylbutanoate-D | 0.00 ± 0.00a | 0.00 ± 0.00a | 0.00 ± 0.00a | 0.00 ± 0.00a | 34.86 ± 0.69A | 19.02 ± 0.99B | 9.92 ± 1.18C | 7.04 ± 0.73D |
| 23 | Ethyl butanoate-M | 0.07 ± 0.00a | 0.05 ± 0.00d | 0.08 ± 0.00a | 0.06 ± 0.00b | 135.76 ± 3.98C | 137.32 ± 3.32C | 153.77 ± 5.91A | 145.70 ± 2.66B |
| 24 | Ethyl butanoate-D | 0.49 ± 0.02a | 0.20 ± 0.00d | 0.26 ± 0.02c | 0.30 ± 0.00b | 730.47 ± 2.11A | 423.36 ± 7.47B | 429.95 ± 3.39B | 307.53 ± 7.79C |
| 25 | Methyl 3-methylbutanoate-M | 0.03 ± 0.00b | 0.04 ± 0.00a | 0.01 ± 0.00c | 0.02 ± 0.00c | 45.84 ± 0.67C | 34.74 ± 0.90D | 57.94 ± 1.08A | 52.77 ± 1.22B |
| 26 | Methyl 3-methylbutanoate-D | 0.08 ± 0.0c | 0.17 ± 0.00b | 0.38 ± 0.02a | 0.38 ± 0.00a | 83.53 ± 2.42S | 28.24 ± 0.61D | 414.97 ± 2.53A | 325.43 ± 7.38B |
| 27 | Methyl butanoate-M | 0.06 ± 0.01a | 0.05 ± 0.00b | 0.05 ± 0.00b | 0.05 ± 0.00b | 127.53 ± 3.32A | 88.54 ± 4.41D | 118.72 ± 4.04B | 107.87 ± 1.44C |
| 28 | Methyl butanoate-D | 0.59 ± 0.02b | 0.72 ± 0.01a | 0.43 ± 0.02d | 0.47 ± 0.01c | 296.47 ± 5.87B | 698.64 ± 9.49A | 221.27 ± 2.15D | 247.69 ± 3.86C |
| 29 | Propyl acetate-D | 0.00 ± 0.00 | 0.00 ± 0.00 | 0.00 ± 0.00 | 0.00 ± 0.00 | 38..47 ± 15.72A | 15.71 ± 0.31C | 35.73 ± 1.35B | 16.63 ± 0.40C |
| 30 | Propyl acetate-M | 0.06 ± 0.00a | 0.04 ± 0.00b | 0.02 ± 0.00c | 0.02 ± 0.00c | 18.72 ± 0.23A | 17.73 ± 0.89A | 14.64 ± 0.36B | 14.73 ± 1.19B |
| 31 | Ethyl propanoate-D | 0.01 ± 0.00c | 0.00 ± 0.00c | 0.01 ± 0.00b | 0.02 ± 0.00a | 268.01 ± 2.81A | 147.31 ± 2.88D | 163.14 ± 4.32C | 196.30 ± 3.65B |
| 32 | Ethyl propanoate-M | 0.01 ± 0.00b | 0.00 ± 0.00b | 0.01 ± 0.00a | 0.01 ± 0.00a | 9.76 ± 0.95C | 15.00 ± 0.18B | 16.21 ± 1.02A | 10.88 ± 0.30C |
| 33 | Isopropyl acetate | 0.02 ± 0.00d | 0.20 ± 0.00a | 0.04 ± 0.00c | 0.06 ± 0.00b | 8.78 ± 0.62C | 14.73 ± 1.80A | 9.93 ± 0.60C | 13.80 ± 2.54B |
| 34 | Ethyl acetate-D | 0.26 ± 0.01b | 0.15 ± 0.0c | 0.28 ± 0.01a | 0.27 ± 0.00ab | 2,017.92 ± 9.69A | 1,566.82 ± 20.40C | 1,958.76 ± 43.35B | 1,556.13 ± 23.78C |
| 35 | Ethyl acetate-M | 0.03 ± 0.00a | 0.02 ± 0.00b | 0.02 ± 0.00b | 0.02 ± 0.00b | 37.01 ± 1.11D | 49.16 ± 0.79B | 44.34 ± 0.55C | 62.72 ± 1.10A |
| 36 | Methyl acetate | 0.06 ± 0.01d | 0.35 ± 0.01b | 0.48 ± 0.02a | 0.40 ± 0.01b | 134.67 ± 3.36D | 331.55 ± 5.71C | 697.06 ± 14.51A | 358.20 ± 4.42B |
| 37 | Methyl propanoate-D | 0.00 ± 0.00b | 0.02 ± 0.00a | 0.02 ± 0.00a | 0.02 ± 0.00a | 26.98 ± 3.48B | 20.82 ± 0.69C | 33.51 ± 0.60A | 19.84 ± 0.53C |
| 38 | Methyl propanoate-M | 0.04 ± 0.00a | 0.03 ± 0.00b | 0.04 ± 0.01a | 0.04 ± 0.00a | 163.07 ± 10.11C | 172.90 ± 3.14BC | 176.84 ± 3.30AB | 187.32 ± 6.22A |
| 39 | Ethyl 2-methylpropanoate-D | 0.00 ± 0.00b | 0.00 ± 0.00b | 0.01 ± 0.00a | 0.01 ± 0.00a | 56.05 ± 1.11A | 9.52 ± 0.66C | 35.16 ± 0.64B | 6.91 ± 0.72D |
| 40 | Ethyl 2-methylpropanoate-M | 0.01 ± 0.00a | 0.01±0.00a | 0.01±0.00a | 0.01±0.00a | 91.96 ± 3.87A | 84.37 ± 1.30B | 94.45 ± 2.67A | 76.97 ± 1.89C |
| 41 | Methyl 2-methylbutanoate | 0.00 ± 0.00c | 0.14 ± 0.00a | 0.06 ± 0.00b | 0.06 ± 0.00b | 132.43 ± 3.59A | 28.73 ± 0.29D | 101.81 ± 1.23B | 51.37 ± 1.94C |
| 42 | Isopropyl butanoate | 0.01 ± 0.00b | 0.07 ± 0.00a | 0.01 ± 0.00b | 0.01 ± 0.00b | 11.56 ± 0.25A | 10.66 ± 1.41AB | 11.82 ± 0.64A | 9.50 ± 0.69B |
| 43 | Butyl hexanoate | 0.04 ± 0.00a | 0.03 ± 0.00b | 0.03 ± 0.00b | 0.04 ± 0.00a | 35.68 ± 1.58 | 36.51 ± 1.60 | 37.67 ± 3.50 | 33.67 ± 3.02 |
| 44 | 3-Methylbutyl 3-methylbutanoate | 0.01 ± 0.00c | 0.01 ± 0.00c | 0.03 ± 0.00b | 0.05 ± 0.00a | 29.03 ± 3.11A | 26.15 ± 3.36B | 26.37 ± 0.43B | 24.83 ± 3.71BC |
| 45 | Butyl 2-methylpropanoate | 0.04 ± 0.00a | 0.01 ± 0.00c | 0.02 ± 0.00b | 0.02 ± 0.00b | 18.74 ± 0.87B | 17.72 ± 1.69B | 22.94 ± 1.63A | 18.89 ± 1.77B |
| 46 | (2-Methylpropyl) acetate | 0.00 ± 0.00a | 0.00 ± 0.00a | 0.00 ± 0.00a | 0.00 ± 0.00a | 60.89 ± 0.19B | 23.56 ± 0.95C | 119.35 ± 0.91A | 10.92 ± 0.68D |
| Total (Esters) | | 3.98 ± 0.17c | 4.53 ± 0.10b | 5.06 ± 0.25a | 4.81 ± 0.03ab | 8,154.75 ± 63.67B | 7,406.27 ± 74.39C | 9,209.64 ± 60.30A | 6,098.89 ± 95.35D |
| **Alcohols (10)** | | | | | | | | | |
| 47 | 1-Hexanol-M | 0.02 ± 0.00d | 0.07 ± 0.01b | 0.05 ± 0.00c | 0.13 ± 0.09a | 768.35 ± 30.29A | 622.85 ± 13.99C | 604.74 ± 14.76C | 717.39 ± 7.90B |
| 48 | 1-Hexanol-D | 0.01 ± 0.00c | 0.02 ± 0.00b | 0.02 ± 0.00b | 0.03 ± 0.00a | 1,261.23 ± 22.03A | 493.47 ± 50.42D | 577.65 ± 3.81C | 765.09 ± 11.19B |
| 49 | 3-Methyl-1-butanol | 0.05 ± 0.00b | 0.04 ± 0.00c | 0.07 ± 0.00a | 0.08 ± 0.01a | 396.91 ± 2.96C | 456.50 ± 6.23A | 397.38 ± 4.68C | 427.41 ± 7.29B |
| 50 | 1-Butanol-M | 0.01 ± 0.00a | 0.01 ± 0.00a | 0.01 ± 0.00a | 0.01 ± 0.00a | 68.97 ± 4.59B | 37.24 ± 1.82D | 44.59 ± 1.34C | 80.13 ± 0.16A |
| 51 | 1-Butanol-D | 0.00 ± 0.00a | 0.00 ± 0.00a | 0.00 ± 0.00a | 0.00±0.00a | 80.06 ± 3.30A | 12.86 ± 0.93D | 28.50 ± 1.34C | 55.59 ± 0.16B |
| 52 | Ethanol | 0.30 ± 0.02a | 0.26 ± 0.01b | 0.28± 0.02ab | 0.21 ± 0.01c | 1,811.94 ± 26.74A | 1,699.97 ± 23.78C | 1,754.55 ± 21.44B | 1,599.01 ± 14.59D |
| 53 | 2-Methyl-1-propanol-M | 0.00 ± 0.00a | 0.00 ± 0.00a | 0.00 ± 0.00a | 0.00 ± 0.00a | 91.43 ± 2.04B | 137.50 ± 4.00A | 94.15 ± 2.96B | 133.40 ± 1.82A |
| 54 | 2-Methyl-1-propanol-D | 0.03 ± 0.00b | 0.04 ± 0.00a | 0.02 ± 0.00c | 0.02 ± 0.00c | 1,322.71 ± 10.34A | 910.08 ± 5.79C | 1,313.35 ± 12.03A | 975.84 ± 6.12B |
| 55 | 1-Propanol-M | 0.01 ± 0.00a | 0.01 ± 0.00a | 0.01 ± 0.00a | 0.01 ± 0.00a | 102.19 ± 1.39C | 100.54 ± 1.26C | 110.87 ± 1.01B | 121.76 ± 0.50A |
| 56 | 1-Propanol-D | 0.01 ± 0.0 | 0.02 ± 0.00 | 0.02 ± 0.00 | 0.01 ± 0.00 | 227.95 ± 7.05D | 239.60 ± 1.27C | 321.01 ± 1.69B | 389.37 ± 1.06A |
| Total (Alcohols) | | 0.45 ± 0.02b | 0.46± 0.00ab | 0.47 ± 0.02ab | 0.50 ± 0.01a | 6,131.72 ± 68.54A | 4,710.62 ± 94.09C | 5,246.80 ± 41.04B | 5,265.00 ± 11.64B |
| **Aldehydes (16)** | | | | | | | | | |
| 57 | (E)-Hex-2-enal-M | 0.18 ± 0.01a | 0.18 ± 0.00a | 0.14 ± 0.00c | 0.13 ± 0.00d | 67.51 ± 19.35A | 53.65 ± 6.71AB | 44.61 ± 0.58B | 47.86 ± 3.81AB |
| 58 | (E)-Hex-2-enal-D | 0.95 ± 0.04a | 0.84 ± 0.02b | 0.99 ± 0.04a | 0.89 ± 0.01b | 398.99 ± 18.85A | 236.18 ± 16.58C | 303.11 ± 18.61B | 163.60 ± 4.33D |
| 59 | (E)-2-Pentenal-M | 0.06 ± 0.00a | 0.03 ± 0.00d | 0.05 ± 0.00b | 0.04 ± 0.00c | 15.96 ± 1.24A | 7.98 ± 1.34C | 8.71 ± 1.27C | 12.97 ± 0.45B |
| 60 | (E)-2-Pentenal-D | 0.07 ± 0.00c | 0.02 ± 0.00d | 0.11 ± 0.01a | 0.10 ± 0.00b | 12.76 ± 3.09A | 11.31 ± 1.67A | 13.64 ± 1.48A | 7.82 ± 0.30AB |
| 61 | 1-Hexanal-M | 0.26 ± 0.01a | 0.23 ± 0.01b | 0.18 ± 0.01c | 0.17 ± 0.01c | 612.36 ± 9.69A | 430.87 ± 7.76B | 594.17 ± 15.58A | 445.69 ± 8.71B |
| 62 | 1-Hexanal-D | 0.95 ± 0.04b | 0.79 ± 0.03c | 0.96 ± 0.03b | 1.02 ± 0.02a | 101.21 ± 11.55A | 92.93 ± 6.04AB | 105.77 ± 11.77A | 88.20 ± 2.77B |
| 63 | 1-Propanal-M | 0.03 ± 0.00b | 0.03 ± 0.00b | 0.03 ± 0.00b | 0.04 ± 0.00a | 33.36 ± 2.27C | 80.83 ± 1.24A | 66.33 ± 1.52B | 79.71 ± 3.65A |
| 64 | 1-Propanal-D | 0.11 ± 0.00c | 0.06 ± 0.00d | 0.14 ± 0.00a | 0.13 ± 0.00b | 36.41 ± 8.64AB | 29.50 ± 3.39B | 28.44 ± 0.26B | 39.46 ± 2.73A |
| 65 | 2-Methylpropanal | 0.02 ± 0.00c | 0.02 ± 0.00c | 0.03 ± 0.00b | 0.04 ± 0.00a | 24.51 ± 0.95C | 26.02 ± 1.14C | 48.74 ± 0.97B | 51.77 ± 1.39A |
| 66 | (E)-2-Octenal | 0.06 ± 0.00c | 0.04 ± 0.00d | 0.08 ± 0.01b | 0.11 ± 0.00a | 53.05 ± 4.59A | 37.69 ± 6.70B | 46.48 ± 6.34B | 42.31 ± 3.65B |
| 67 | (E)-2-Heptenal-M | 0.07 ± 0.00c | 0.04 ± 0.00d | 0.10 ± 0.00b | 0.12 ± 0.00a | 54.62 ± 4.38A | 43.86 ± 0.72B | 53.86 ± 5.09A | 40.53 ± 1.93B |
| 68 | (E)-2-Heptenal-D | 0.01 ± 0.00b | 0.01 ± 0.00b | 0.01 ± 0.00b | 0.02 ± 0.00a | 12.60 ± 1.61A | 10.58 ± 1.91B | 10.27 ± 0.70B | 12.08 ± 2.34A |
| 69 | 2-Hexenal-M | 0.10 ± 0.00a | 0.08 ± 0.00c | 0.10 ± 0.00a | 0.09 ± 0.00b | 40.86 ± 0.21 | 41.36 ± 1.86 | 40.35 ± 2.74 | 42.82 ± 1.24 |
| 70 | 2-Hexenal-D | 0.07 ± 0.00c | 0.05 ± 0.00d | 0.13 ± 0.00a | 0.12 ± 0.00b | 2,281.93 ± 26.04A | 2,012.39 ± 18.24B | 2,264.39 ± 18.18A | 1,917.49 ± 11.00C |
| 71 | 3-Methylbutanal-M | 0.03 ± 0.00a | 0.03 ± 0.00a | 0.02 ± 0.00b | 0.02 ± 0.00b | 79.81 ± 6.17A | 79.02 ± 1.51A | 57.24 ± 1.95B | 59.40 ± 1.45B |
| 72 | 3-Methylbutanal-D | 0.05 ± 0.00b | 0.02 ± 0.00c | 0.05 ± 0.00b | 0.08 ± 0.00a | 7.50 ± 1.87C | 12.09 ± 0.73B | 13.39 ± 0.43B | 16.21 ± 0.85A |
| Total (Aldehydes) | | 3.03 ± 0.11a | 2.47 ± 0.06b | 3.13 ± 0.08a | 3.09 ± 0.06a | 3,833.47 ± 60.39A | 3,206.29 ± 64.76C | 3,699.50 ± 70.14B | 3,067.92 ± 26.89D |
| **Ketones (12)** | | | | | | | | | |
| 73 | 3-Pentanone | 0.06 ± 0.00b | 0.02 ± 0.00d | 0.05 ± 0.00c | 0.09 ± 0.00a | 271.04 ± 2.56A | 112.94 ± 2.95D | 235.28 ± 1.36B | 194.42 ± 1.84C |
| 74 | 2-Propanone | 0.32 ± 0.00a | 0.24 ± 0.00b | 0.10 ± 0.00d | 0.17 ± 0.00c | 913.60 ± 7.01A | 720.61 ±9.65B | 390.90 ± 2.75D | 453.66 ± 3.54C |
| 75 | 4-Methyl-2-pentanone-D | 0.17 ± 0.01a | 0.08 ± 0.00c | 0.04 ± 0.00d | 0.09 ± 0.01b | 298.91 ± 6.10A | 32.64 ± 2.02D | 73.51 ± 1.63C | 113.27 ± 1.30B |
| 76 | 4-Methyl-2-pentanone-M | 0.04 ± 0.00a | 0.01 ± 0.00d | 0.02 ± 0.00c | 0.03 ± 0.00b | 14.78 ± 0.82B | 13.95 ± 1.15B | 10.68 ± 0.25C | 19.11 ± 0.86A |
| 77 | 2-Heptanone-M | 0.07 ± 0.00a | 0.03 ± 0.00b | 0.03 ± 0.00b | 0.03 ± 0.00b | 66.26 ± 3.30A | 39.49 ± 1.12B | 26.41 ± 1.17C | 18.18 ± 0.28D |
| 78 | 2-Heptanone-D | 0.05 ± 0.00a | 0.01 ± 0.00b | 0.01 ± 0.00b | 0.01 ± 0.00b | 33.54 ± 0.82A | 11.49 ± 0.59B | 5.97 ± 0.05C | 5.86 ± 1.16C |
| 79 | 1-Penten-3-one-M | 0.04 ± 0.00a | 0.03 ± 0.00b | 0.02 ± 0.00c | 0.02 ± 0.00c | 75.05 ± 3.40A | 67.97 ± 1.01B | 64.87 ± 2.16B | 66.87 ± 0.66B |
| 80 | 1-Penten-3-one-D | 0.17 ± 0.00a | 0.05 ± 0.00d | 0.10 ± 0.01b | 0.07 ± 0.00c | 21.54 ± 0.35C | 18.78 ± 1.00D | 49.63 ± 1.09A | 41.04 ± 1.34B |
| 81 | 2-Butanone-M | 0.02 ± 0.00a | 0.01 ± 0.00b | 0.01 ± 0.00b | 0.01 ± 0.00b | 10.24 ± 1.55C | 19.21 ± 0.84B | 14.25 ± 1.95C | 22.46 ± 3.76A |
| 82 | 2-Butanone-D | 0.02 ± 0.00a | 0.01 ± 0.00b | 0.01 ± 0.00b | 0.00 ± 0.00c | 5.82 ± 0.80B | 7.78 ± 0.81A | 4.82 ± 0.27BC | 4.19 ± 0.47C |
| 83 | 3-Penten-2-one-M | 0.01± 0.00a | 0.00 ± 0.00b | 0.00 ± 0.00b | 0.00 ± 0.00b | 67.93 ± 0.40B | 40.57 ± 2.30C | 92.12 ± 2.16A | 26.93 ± 0.82D |
| 84 | 3-Penten-2-one-D | 0.00 ± 0.00a | 0.00 ± 0.00a | 0.00 ± 0.00a | 0.00 ± 0.00a | 44.40 ± 1.65B | 23.79 ± 0.60C | 72.13 ± 1.46A | 12.42 ± 0.90D |
| Total (Ketones) | | 0.96 ± 0.03a | 0.48 ± 0.01b | 0.39 ± 0.01c | 0.51 ± 0.02b | 1,823.12 ± 14.80A | 1,109.22 ± 11.92B | 1,040.57 ± 5.13C | 978.41 ± 6.89D |
| **Acids (2)** | | | | | | | | | |
| 85 | Acetic acid-M | 0.17 ± 0.03a | 0.11 ± 0.01b | 0.09 ± 0.00c | 0.08 ± 0.00c | 354.99 ± 23.33A | 313.07 ± 16.90AB | 338.53 ± 26.98AB | 301.96 ± 9.42B |
| 86 | Acetic acid-D | 0.02 ± 0.01a | 0.01 ± 0.00b | 0.01 ± 0.00b | 0.01 ± 0.00b | 40.83 ± 2.88A | 31.47 ± 1.61C | 37.60 ± 0.62B | 28.45 ± 0.42C |
| Total (Acids) | | 0.19 ± 0.04a | 0.13 ± 0.01b | 0.10 ± 0.00b | 0.09 ± 0.00b | 395.82 ± 31.17A | 344.54 ± 17.75BC | 376.13 ± 27.60B | 330.41 ± 9.00C |
| **Terpenes (2)** | | | | | | | | | |
| 87 | (RS)-Linalool | 0.02 ± 0.00c | 0.02 ± 0.00c | 0.03 ± 0.00b | 0.04 ± 0.00a | 53.30 ± 2.24A | 46.24 ± 4.32B | 59.53 ± 3.94A | 58.17 ± 2.41A |
| 88 | α-Terpinolene | 0.06 ± 0.00b | 0.00 ± 0.00d | 0.08 ± 0.00a | 0.01 ± 0.00c | 61.76 ± 3.15B | 22.97 ± 3.09C | 74.97 ± 1.87A | 20.94 ± 2.32C |
| Total (Terpenes) | | 0.08 ± 0.00b | 0.02 ± 0.00d | 0.11 ± 0.00a | 0.05 ± 0.00c | 115.07 ± 4.78B | 69.21 ± 2.61D | 134.50 ± 3.88A | 79.11 ± 3.52C |
| **Total** | | 8.80 ± 0.35b | 8.28 ± 0.03c | 9.36 ± 0.35a | 9.22 ± 0.10ab | 20,953.95 ± 173.69A | 17,346.16 ± 255.32C | 20,207.13 ± 178.70B | 16,319.74 ± 126.17D |

Note: Different lowercase letters indicate significant differences among strawberry samples across harvest dates, while different uppercase letters indicate significant differences among wine samples across harvest dates, according to Duncan's test (*p* < 0.05).

**Table S8. Significance values of two-way ANOVA for the effects of cultivar, HAR_T_ and their interaction on key volatile profiles of different strawberries and wines**

| **NO**. | V**olatiles** | **Strawberry fruits** | | | V**olatiles** | **Strawberry wines** | | |
| --- | --- | --- | --- | --- | --- | --- | --- | --- |
|  |  | **Cultivar (C)** | **HAR_T_** | **C × HAR_T_** |  | **Cultivar (C)** | **HAR_T_** | **C × HAR_T_** |
|  |  |  |  |  |  |  |  |  |
| 1 | Ethanol | ***** | ****** | ****** | 1-Butanol | ****** | ****** | ****** |
| 2 | (E)-Hex-2-en-1-yl acetate | ****** | ****** | ***** | 3-Methylbutyl acetate | ****** | ****** | ****** |
| 3 | Hexyl acetate | ****** | ****** | ****** | Hexyl acetate | ****** | ****** | ****** |
| 4 | 1-Hexanol | ****** | ****** | ****** | Methyl acetate | ****** | ****** | ****** |
| 5 | (2-Methylpropyl) acetate | ****** | ****** | n**s** | 1-Hexanol | ****** | ***** | ****** |
| 6 | Methyl hexanoate | ****** | ****** | ****** | Ethyl propanoate | ****** | ****** | ****** |
| 7 | 2-Methylpropanal | ****** | ****** | n**s** | Methyl hexanoate | ****** | ****** | ****** |
| 8 | (E)-Hex-2-enal | ****** | ****** | ****** | (2-Methylpropyl) acetate | ****** | ****** | ****** |
| 9 | Pentyl acetate | ****** | ****** | ****** | Ethyl hexanoate | ****** | ****** | ****** |
| 10 | 3-Pentanone | ****** | ***** | ****** | Pent-3-en-2-one | ****** | ****** | ****** |
| 11 | Butyl hexanoate | ***** | n**s** | ****** | 4-Methylpentan-2-one | ****** | ****** | ****** |
| 12 | Pent-1-en-3-one | ****** | ****** | ****** | Methyl butanoate | ****** | ****** | ****** |
| 13 | Butyl 2-methylpropanoate | ****** | ****** | ****** | Ethyl 3-methylbutanoate | ****** | ***** | ****** |
| 14 | α-Terpinolene | n**s** | n**s** | ****** | Butyl acetate | ****** | ****** | ***** |
| 15 | 4-Methylpentan-2-one | ****** | ****** | ****** | Methyl 3-methylbutanoate | ****** | ****** | ***** |
| 16 | Isopropyl acetate | ****** | ****** | ***** | 2-Propanone | ****** | ****** | ****** |
| 17 | 3-Methylbutanal | ****** | ***** | ****** | 1-Propanol | ****** | ****** | ****** |
| 18 |  |  |  |  | Pent-1-en-3-one | ****** | ***** | n**s** |
| 19 |  |  |  |  | 2-Methylpropanal | ****** | ****** | ***** |
| 20 |  |  |  |  | 3-Methylbutanal | ****** | ****** | n**s** |
| 21 |  |  |  |  | Ethyl pentanoate | ***** | ****** | ****** |
| 22 |  |  |  |  | 3-Pentanone | ****** | ****** | ****** |
| 23 |  |  |  |  | Ethyl 2-methylbutanoate | ****** | ****** | ****** |
| 24 |  |  |  |  | Ethanol | ****** | ****** | ****** |
| 25 |  |  |  |  | α-Terpinolene | ****** | ****** | ****** |
| 26 |  |  |  |  | 2-Hexenal | ****** | ****** | ****** |
| 27 |  |  |  |  | 2-Heptanone | ****** | ****** | ****** |

Note: Data were analyzed by two-way ANOVA with cultivar (C) and harvest time (HAR_T_) as fixed factors. Significance levels: ns, not significant (*p* ≥ 0.05); *, *p* < 0.05; **, *p* < 0.01. Each treatment was analyzed with three biological replicates.

**Fig. S1. Permutation tests of the GC-IMS based OPLS-DA models for different strawberries (A) and wines (B).**
